# Dectin-1 Positive Dendritic Cells Expand after Infection with *Leishmania major* Parasites and Represent Promising Targets for Vaccine Development

**DOI:** 10.3389/fimmu.2018.00263

**Published:** 2018-02-26

**Authors:** Nicole Zimara, Menberework Chanyalew, Abraham Aseffa, Ger van Zandbergen, Bernd Lepenies, Maximilian Schmid, Richard Weiss, Anne Rascle, Anja Kathrin Wege, Jonathan Jantsch, Valentin Schatz, Gordon D. Brown, Uwe Ritter

**Affiliations:** ^1^Regensburg Center for Interventional Immunology (RCI), Institute of Immunology, University Medical Center Regensburg, University of Regensburg, Regensburg, Germany; ^2^Armauer Hansen Research Institute, Leishmaniasis Research Laboratory, Addis Ababa, Ethiopia; ^3^Federal Institute for Vaccines and Biomedicines, Division of Immunology, Paul Ehrlich Institute, Langen, Germany; ^4^University of Veterinary Medicine Hannover, Immunology Unit, Research Center for Emerging Infections and Zoonoses (RIZ), Hannover, Germany; ^5^Department of Internal Medicine III, Hematology and Oncology, University Hospital Regensburg, Regensburg, Germany; ^6^Department of Molecular Biology, Division of Allergy and Immunology, University of Salzburg, Salzburg, Austria; ^7^Department of Gynecology and Obstetrics, University Medical Center Regensburg, Regensburg, Germany; ^8^Institute of Clinical Microbiology and Hygiene, University Hospital of Regensburg, University of Regensburg, Regensburg, Germany; ^9^MRC Centre for Medical Mycology, University of Aberdeen, Aberdeen, United Kingdom

**Keywords:** Curdlan, β-glucan, Dectin-1, cutaneous leishmaniasis, adaptive immunity, dendritic cells, T helper 1 and T helper 2 cells

## Abstract

Resistant mouse strains mount a protective T cell-mediated immune response upon infection with *Leishmania* (L.) parasites. Healing correlates with a T helper (Th) cell-type 1 response characterized by a pronounced IFN-γ production, while susceptibility is associated with an IL-4-dependent Th2-type response. It has been shown that dermal dendritic cells are crucial for inducing protective Th1-mediated immunity. Additionally, there is growing evidence that C-type lectin receptor (CLR)-mediated signaling is involved in directing adaptive immunity against pathogens. However, little is known about the function of the CLR Dectin-1 in modulating Th1- or Th2-type immune responses by DC subsets in leishmaniasis. We characterized the expression of Dectin-1 on CD11c^+^ DCs in peripheral blood, at the site of infection, and skin-draining lymph nodes of *L. major*-infected C57BL/6 and BALB/c mice and in peripheral blood of patients suffering from cutaneous leishmaniasis (CL). Both mouse strains responded with an expansion of Dectin-1^+^ DCs within the analyzed tissues. In accordance with the experimental model, Dectin-1^+^ DCs expanded as well in the peripheral blood of CL patients. To study the role of Dectin-1^+^ DCs in adaptive immunity against *L. major*, we analyzed the T cell stimulating potential of bone marrow-derived dendritic cells (BMDCs) in the presence of the Dectin-1 agonist Curdlan. These experiments revealed that Curdlan induces the maturation of BMDCs and the expansion of *Leishmania*-specific CD4^+^ T cells. Based on these findings, we evaluated the impact of Curdlan/Dectin-1 interactions in experimental leishmaniasis and were able to demonstrate that the presence of Curdlan at the site of infection modulates the course of disease in BALB/c mice: wild-type BALB/c mice treated intradermally with Curdlan developed a protective immune response against *L. major* whereas Dectin-1^−/−^ BALB/c mice still developed the fatal course of disease after Curdlan treatment. Furthermore, the vaccination of BALB/c mice with a combination of soluble *L. major* antigens and Curdlan was able to provide a partial protection from severe leishmaniasis. These findings indicate that the ligation of Dectin-1 on DCs acts as an important checkpoint in adaptive immunity against *L. major* and should therefore be considered in future whole-organism vaccination strategies.

## Introduction

Cutaneous leishmaniasis (CL) is a vector-borne parasitic infectious disease encountered in tropical and subtropical regions of the world (1). The causative agents are flagellated protozoans of the genus *Leishmania (L.)*, which are inoculated into the skin during the blood meal of female sandflies. Different clinical manifestations in humans, ranging from a self-limiting cutaneous infection to disseminating visceral leishmaniasis (VL), are described with respect to the transmitting vectors and *Leishmania* species (2).

Comparable to the course of disease in humans, *L. major* parasites can develop cutaneous manifestations in C57BL/6 and BALB/c mouse models (3). The infection of inbred mice with stationary phase promastigote *Leishmania* parasites allowed the examination of basic mechanisms, resulting in innate and adaptive T cell-mediated immunity (3).

It is known that *L. major* parasites require phagocytic cells for replication and spreading within the host (4). In this regard, neutrophils and macrophages play a pivotal role as host cells for the initial survival and spreading of parasites. However, macrophages produce leishmanicidal molecules after appropriate activation by certain T helper (Th) 1 cytokines such as IFN-γ (3, 5) and become effector cells during the host response against *L. major*. Thus, healing of CL in C57BL/6 mice is associated with a pronounced expansion of IFN-γ-secreting Th1 cells (6), whereas susceptibility of BALB/c mice is associated with a diminished IFN-γ, but increased IL-4-production in high-dose infection models (5, 7, 8). Therefore, CD4^+^ T cells are crucial key players in modulating antigen-specific immunity in leishmaniasis (3).

It is mostly accepted that Th1- and Th2-effector cells derive from a common CD4^+^ T cell precursor (9). Several stimuli have been reported to influence the pathway of maturation of these CD4^+^ T cell progenitors (10). Using CD4^+^ T cells, transgenic for a unique TCR alpha/beta receptor, it was proven that distinct cytokines such as IL-12 and IL-4 are crucial for the polarization of Th1 and Th2 cells (5, 10). Th1 cell differentiation takes place after *Leishmania*-specific T cells have been primed sufficiently by antigen-presenting cells in skin-draining lymph nodes (SDLNs) (11). Based on the current model it must be assumed that Langerin^−^ dermal dendritic cells (dDCs) are pivotal for the induction of the protective Th1-mediated immune response against *L. major* in C57BL/6 mice (12–14). Of note, Langerin^+^ epidermal Langerhans cells are dispensable for the generation of protective immunity in experimental leishmaniasis (13–16).

T cell-mediated immunity against *L*. major parasites is a multistep process whereby mature DC subsets are pivotal to start the adaptive immune response within SDLNs (12, 14). Maturation of DCs is accompanied by high expression of chemokine receptor (CCR) 7, paving the way to the SDLN and costimulatory molecules crucial for DC/T-cell communication (17). Certain cytokines such as TNF-α and IL-6 are known to support this process (17, 18). Additionally, pattern recognition receptors (PRRs) binding pathogen components are also potent activators of DC maturation (19). Especially toll-like receptors that represent the most popular PRRs (20), recognize pathogen-associated molecular patterns derived from various pathogens, including viruses, bacteria, fungi, parasites, and protozoa, and are crucial for innate immune mechanisms (21).

The C-type lectin receptors (CLRs) also belong to PRRs and recognize predominantly carbohydrates and non-carbohydrates through mechanisms that are still not fully understood (22). The CLR Dectin-1 recognizes β-glucans (22–25) that naturally occur in the cell wall of fungi (23). In addition to pathogen-derived factors, endogenous *N*-glycans present on the surface of tumor cells also represent potential Dectin-1 ligands and trigger tumor killing by NK cells (26).

Mouse Dectin-1 is expressed by various immunologically relevant cells belonging to the adaptive and innate immune system. Preferentially, monocytes, macrophages, neutrophils, and a subset of T cells were described to be positive for Dectin-1 (27). There is growing evidence that engagement of Dectin-1 on DC subsets is crucial for T-cell polarization in experimental mouse models (28, 29). In humans, Dectin-1 is widely expressed by all monocyte populations as well as by macrophages, DC, neutrophils, eosinophils, and B cells (27, 30). In line with these findings, it was shown that the function of human Dectin-1 is equivalent to that of mouse Dectin-1 (30). Thus, CLRs in general and Dectin-1 in particular might be considered as important checkpoints for adaptive immune responses.

It could be demonstrated that Dectin-1^−/−^ macrophages from C57BL/6 mice show a slightly reduced capacity for phagocytosis of *L. infantum* parasites *in vitro* (31). Thus, Dectin-1 might be involved in the formation of parasitophorous vacuoles (32). In line with these findings, it is important to mention that infected macrophages from C57BL/6 show an enhanced expression of Dectin-1 after infection with *L. amazonensis in vitro* (33). Consequently, the pronounced Dectin-1 expression by infected myeloid cells might potentiate the uptake of parasites and favors the spreading of the obligatory intracellular parasites during the first stage of innate immunity. An interaction of Dectin-1 with parasite-derived carbohydrates was not identified so far. Nevertheless, β-glucan can activate infected macrophages from BALB/c mice to control the replication of *L. donovani* parasites *in vitro* (34, 35). Additionally, it was shown that NK cells can also be activated by *Aureobasidium*-derived soluble branched (1,3–1,6)-β-glucan that results in enhanced cellular immunity against *L. amazonensis* parasites in BALB/c mice (36). The scientific evidence, that β-glucan can modulate innate immune mechanisms against *L. major* parasites at the site of infection, is still pending.

Dectin-1 signaling is also discussed to be crucial in directing adaptive T cell-mediated immune responses. Thus far, it is known that Dectin-1 ligation by fungal components triggers Th1- and Th17-mediated immune responses against fungi (37–41). Accordingly, Dectin-1 deficiency results in impaired T cell-mediated immunity and loss of control of fungal infection (42). Long before Dectin-1 was described as a receptor for β-glucans, these glucose polysaccharides were used as adjuvants for immunization and systemic therapies of VL in BALB/c and C57BL/6 mice (43–47). In line with this, Ghosh et al. were able to efficiently treat BALB/c mice infected with *L. donovani* by multiple intraperitoneal (i.p.) applications of the linear β-glucan Curdlan, which induced Th17-mediated adaptive immunity and macrophage activation (34). Most of the studies investigating the effect of β-glucans were carried out using VL-causing *L. donovani* parasites. However, one study is published demonstrating that multiple systemic applications (i.p. and i.v.) of β-glucan after infection of BALB/c mice with *L. major* parasites blocked lesion development or parasite spreading in normally susceptible BALB/c mice (48). Whether Dectin-1 is responsible for the observed immunological phenomenon has not been shown until now. Furthermore, quantification and characterization of Dectin-1^+^ DCs in experimental leishmaniasis and in patients suffering from CL are missing.

In this study, we investigated the potential impact of β-glucan and of Dectin-1 on DC physiology and subsequent modulation of T-cell immunity. Here, we were able to demonstrate an expansion of Dectin-1^+^ DCs in experimental leishmaniasis as well as in patients suffering from CL. Additional studies revealed that intradermal application of *L. major* parasites in combination with Curdlan changes the course of leishmaniasis: BALB/c mice treated with Curdlan developed a protective immune response against *L. major*, whereas Dectin-1^−/−^ BALB/c mice still suffered from a fatal course of disease after Curdlan treatment. Based on these data, it appears that Dectin-1/Curdlan interactions *per se* are sufficient to modulate Th-cell differentiation. Further *in vitro* studies were performed to explore the cellular mechanisms. One important finding was the change in the phenotype and functionality of infected DCs triggered by Curdlan. They increase the expression of Dectin-1 and costimulatory molecules and become potent antigen-presenting cells, capable of accelerating the expansion of *L. major-*specific T cells.

The results presented in this article support the view that Dectin-1^+^ DCs represent promising targets for modulating adaptive T cell-mediated immunity and should therefore be considered in future whole-organism vaccination strategies.

## Materials and Methods

### Human Samples

7–10 mL of peripheral blood samples were collected from patients suffering from CL and Ethiopian healthy controls (EHCs) living in Oromo and Amhara regions of Ethiopia. The sample collection was permitted based on the local ethical committee (allowance number PO25/08, Addis Ababa, Ethiopia) and the national health research ethics review committee (approval number 310/227/07, Addis Ababa). Written informed consent was obtained from the participants of this study. CL was confirmed by positive parasite cultures and PCR analysis as described elsewhere (49). CL patients were excluded from the study if they show one of the following criteria: younger than 18 and older than 55 years, chronic lesions (more then 6 months), positive for HIV, clinical evidence for coinfections, and drug intake.

### Mice

Female wild-type BALB/c and C57BL/6 mice (Janvier Labs, Le Genest St. Isle, France) were kept at the animal facility of the University of Regensburg under pathogen-free conditions. Dectin-1^−/−^ mice on BALB/c background [kindly provided by G.D. Brown, University of Aberdeen (50)] were bred and maintained under conventional animal housing conditions. All experiments and animal housing were performed according to the guidelines for the care and use of experimental animals. The animal work was approved by the local veterinary authorities of the district government based on the European guidelines and national regulations of the German Animal Protection Act (approval no. AZ 54-2532.105/11). Female animals between 6 and 12 weeks of age were used for experiments.

### Parasites and Infection of Mice

Virulent *L. major* parasites (MHOM/IL/81/FE/BNI) were propagated *in vitro* in blood agar cultures as described previously (51). Stationary phase promastigotes from the third to seventh *in vitro* passage were harvested, washed four times, and resuspended in PBS. Mice were infected *via* intradermal injection of 3 × 10^6^ stationary phase promastigotes in 30 µL into the hind footpads. The increase in lesion size was monitored weekly by measuring the footpad thickness with a metric caliper (Kroeplin Schnelltaster, Schlüchtern, Germany). The increase in footpad thickness (%) was determined as described elsewhere (52).

### Curdlan Application

Curdlan (WAKO Chemicals GmbH, Neuss, Germany) was dissolved in sterile PBS to a concentration of 50 µg/µL. 3 × 10^6^ stationary phase promastigotes were resuspended in 30 µL of Curdlan solution and injected intradermally into the hind footpads. Alternatively, soluble *L. major* antigens [SLA; (15)] corresponding to 3 × 10^6^ stationary phase promastigotes were used. The course of disease was monitored as described earlier.

### Quantification of *L. major*-Specific IgG Subtypes

Serum from naïve mice and from mice infected with *L. major* parasites was prepared at the corresponding time point and thereafter stored at −20°C until use. Detection of *L. major*-specific IgG_1_ (Invitrogen, Darmstadt, Germany*)*, IgG_2a_ (BD Pharmingen, Heidelberg), and IgG_2c_ (Jackson ImmunoResearch, Hamburg Germany) isotypes were performed as described earlier (53). The results were standardized by calculation of relative ELISA units (REU). REU were determined by the formula: OD_450_ serum (infected mice)/OD_450_ serum (naïve mice).

### Delayed-Type Hypersensitivity (DTH) Reaction

Three weeks after *L. major* infection, SLA (corresponding to 3 × 10^6^ parasites) was injected in a volume of 20 µL in the foreleg. As a control, 20 µL of PBS was injected in the contra lateral foreleg. The increase in swelling was measured with a metric caliper (Kroeplin Schnelltaster) and referred to the control foreleg. The swelling was measured with a metric caliper (Kroeplin Schnelltaster) after 24, 48, and 72 h.

### Generation of Bone Marrow-Derived Dendritic Cells (BMDCs)

Bone marrow-derived dendritic cells were generated as previously described (54). Bone marrow cells from C57BL/6 or BALB/c mice were seeded in 10 cm BD Tissue culture dishes at a density of 2 × 10^6^ per dish in 10 mL of RPMI medium supplemented with 10% fetal calf serum (FCS; PAN Biotech GmbH), penicillin–streptomycin, 50 µM β-ME, and 10% GM-CSF containing supernatant harvested from Ag8653 myeloma cells transfected with the gene encoding murine GM-CSF (kindly provided by B. Stockinger, NMRI, Mill Hill, London, UK). BMDCs were harvested on day 10 for T cell proliferation experiments.

### Generation of Bone Marrow-Derived Macrophages (BMDMs)

Mouse BMDMs were generated from female BALB/c mice and cultured for 7 days in hydrophobic Teflon bags (FT FEP 100C Dupont, American Durafilm, Holliston, MA) as described earlier (55, 56). For killing experiments, BMDMs were pulse-infected with *L. major* promastigotes at a 1:30 ratio for 4 h as described earlier (56). After infection, extracellular promastigotes were removed by washing with PBS and cultured in the absence or presence of Curdlan (50, 100, and 250 µg/mL) or LPS/IFN-γ (10 and 20 ng/mL, respectively). After 72 h, BMDMs were fixed, stained, and analyzed microscopically using Diff-Quick staining (Medion Diagnostics, Düdingen, Switzerland) for the determination of the percentage of infected cells and the number of parasites per infected cell. LPS (*Escherichia coli* O111:B4) was purchased from Sigma-Aldrich (Taufkirchen, Germany). Recombinant murine IFN-γ was purchased from eBioscience (Frankfurt, Germany).

### Nitrite Accumulation

Nitrite accumulation in the supernatants was determined as an indicator for NO activity after stimulation of BMDMs using the Griess reaction as described earlier (56).

### Labeling of *Leishmania* Parasites with CFSE

Stationary phase promastigotes from the third to seventh *in vitro* passage were harvested, washed four times, and resuspended in PBS. After lysis of the remaining erythrocytes, parasites were labeled by incubation in 1 µM CFSE staining solution as described earlier (57).

### *Leishmania*-Specific T Cell Proliferation Assay

C57BL/6 and BALB/c mice were infected with *L. major* parasites as described earlier. Ten days post infection, SDLNs were removed, and T cells were enriched untouched *via* MACS separation columns (Miltenyi Biotec, Bergisch Gladbach) according to the manufacturer’s guidelines. Single-cell suspensions were labeled with PE-conjugated CD11b, CD11c, and B220 antibodies (Abs) and subsequently detected with anti-PE Beads (Miltenyi Biotec) in order to deplete myeloid cells and B cells. The purified T cells were labeled with CFSE as described earlier (15). 2 × 10^5^ CFSE-labeled T cells were seeded in 96-well round-bottom plates (Nunc) to 2 × 10^4^ BMDCs that have been primed for 24 h with SLA (as an equivalent of 5 parasites: 1 BMDC) or *L. major* parasites (10 parasites: 1 BMDC) in the presence or absence of Curdlan (50 μg/200 μL). 72 h after coculture, the cells were analyzed by flow cytometry. The proliferation index of samples was calculated according to the formula: (number of proliferating cells after stimulation)/(number of proliferating cells in the absence of stimulation). The proliferation index of stimulated samples was then normalized to that of unstimulated BMDC/T-cell cultures. Supernatants were collected and stored at −80°C for cytokine analysis.

### CLR-Fc Fusion Protein Staining of Parasites

The CLR-Fc fusion proteins (Dectin-1, CLEC-9a, and MGL-1) were prepared as described previously (58). After blocking (PBS/10% FCS/10% mouse serum), *L. major* parasites were washed three times in PBS. To analyze interactions with CLRs, parasites were incubated with 20 µg/mL of CLR-Fc fusion proteins diluted in lectin binding buffer (50 mM HEPES, 5 mM MgCl_2_, and 5 mM CaCl_2_, in dH_2_O, pH 7,4) at 4°C for 1 h. After three washing steps with PBS, CLR-Fc binding to *L. major* was detected with a FITC-conjugated goat anti-hFc Ab (Dianova, Hamburg, Germany). Parasite DNA was detected with 6-diamidino-2-phenylindole obtained from Sigma Aldrich, as described earlier (59). After mounting with PermaFluor (Thermo Scientific, Dreieich, Germany), the sections were analyzed using Axio Imager.M1 (Zeiss, Jena, Germany) equipped with high-sensitivity gray scale digital camera (AxioCam MRm, Zeiss). Separate images were collected for each section, analyzed, and merged afterward (acquisition software: Zeiss AxioVision 4.6.3). Final image processing for illustrations was performed using Adobe Photoshop Elements (Adobe Systems GmbH, Munich, Germany).

### Flow Cytometry Analysis of Mouse Samples

Single-cell suspensions of tissues were generated as described elsewhere (60). The following Abs and reagents were used for phenotyping of single-cells: PE-labeled anti-mouse CD11c Ab (clone N418; eBioscience, Frankfurt, Germany), APC-labeled anti-mouse CD11b (clone M1/70, eBioscience, Germany), PE-labeled anti-mouse F4/80 (clone 521204, R&D), biotinylated anti-mouse Dectin-1 Ab (clone 2A11; Bio-Rad AbD Serotec GmbH, Puchheim, Germany), AlexaFluor647-labeled anti-mouse CD86 Ab (clone GL-1; BioLegend, San Diego, CA, USA), and FITC-labeled anti-BrdU Ab (clone B44; BD Pharmingen, Heidelberg, Germany). Depending on the experimental setup, the detection of biotinylated anti-mouse Dectin-1 was performed using streptavidin-conjugated to V500 (BD, Pharmingen, #561419), eFluor450 (eBioscience, #48-4317-82), Pacific Orange (Thermo Fisher, Munich, Germany), or PerCP (BD, Pharmingen, #554064). Ab specificity was verified using appropriate isotype controls and Dectin-1^−/−^ mice (Figure S1 in Supplementary Material). Multicolor flow cytometry was performed as described previously (13). Mice were fed with BrdU-containing drinking water (0.8 mg/mL, Sigma, Deisenhofen, Germany) starting 3 days before the termination of the experiment. BrdU labeling was performed according to the manufacturer’s instructions (BD Pharmingen, Heidelberg, Germany). After labeling of surface epitopes, the cells were fixed and permeabilized with Cytofix/Cytoperm buffer (BD Pharmingen, Heidelberg, Germany) for 30 min on ice and afterward incubated for 10 min on ice with 0.01% Triton X-100 (SERVA, Heidelberg, Germany) in PBS/0.1% BSA (PAN, Aidenbach, Germany). For the detection of incorporated BrdU, the cells were treated with 30 µg DNAse (Sigma, Munich, Germany) for 1 h at 37°C. Labeling of cells with anti-BrdU Ab was performed for 20 min at room temperature. Cells were collected using BD™ LSRII Flow Cytometer (BD Biosciences, Heidelberg, Germany) and analyzed with FlowJo software (Tree Star Inc., Ashland, OR, USA). The determination of the total cell numbers in tissues was performed as described earlier (61).

### Flow Cytometry Analysis of Human Samples

Surface antigens were detected on fresh cells using a modified version of the method described by Aldebert et al. (62). 7–10 mL of blood from patients and EHCs were taken with BD Vacutainer (BD Biosciences, UK) containing 170 IU of lithium heparin. 200 µL of whole blood cells was incubated for 20 min with FcR blocking reagent (Miltenyi Biotec GmbH, Germany). The staining was performed for 30 min at 4°C. The following Abs were used: FITC-labeled anti-lineage (Lin) panel (CD3, CD14, CD16, CD19, CD20, and CD56) (BD), PerCP-labeled anti-HLA-DR (R&D, clone L203), PE-labeled anti-CD123 (R&D, clone 32703), PE-Cy5.5-labeled anti-CD11c (BD, clone B-Ly6), and APC-labeled anti-Dectin-1 (R&D, clone 259931). Erythrocytes were lysed with BD FACS™ Lysing Solution (BD Biosciences), and the remaining white blood cells were washed and resuspended in FACS buffer (PBS buffer, pH 7.2 1% BSA fraction V, Roth, 8076.2), 0.01% NaN_3_ (Sigma: S2002-25G), and 1% human serum. A total of 200,000 events were acquired and the positive population gated using the isotype controls for the different fluorochromes. Data were acquired with a FACS Canto flow cytometer (BD Bioscience). Data analysis was performed with FlowJo 8.8.6.

### Gene Expression Analysis by Quantitative RT-PCR

CD4^+^ T cells were sorted with the CD4^+^ Cell isolation Kit^®^ (Miltenyi Biotec, Bergisch Gladbach, Germany) according to the manufacturer’s specification. Following cell sorting, the CD4^+^ cells were lysed in 350 µL Buffer RA1 (Macherey-Nagel, Düren, Germany) supplemented with 3.5 µL β-ME. Total RNAs were isolated using the NucleoSpin^®^ RNA kit (Macherey-Nagel), following the manufacturer’s instructions. RNAs were quantified by spectrophotometry and 170 ng of RNAs were used for cDNA synthesis using the iScript cDNA Synthesis kit (Bio-Rad Laboratories, München, Germany), as recommended by the manufacturer. Quantitative PCR was performed on a RotorGene Q (Qiagen, Hilden, Germany) using a two-step PCR program (95°C 15 s, 60°C 60 s; 40 cycles). 20 µL quantitative PCR reactions were performed using 0.3–0.6 µL of cDNA template (corresponding to 2.5–5 ng RNA equivalent) and a self-made master-mix containing SYBR Green I and HotStarTaq DNA Polymerase (Qiagen). Data were normalized to GAPDH mRNAs and expressed as relative mRNA levels, as previously reported (63, 64). Forward (fwd) and reverse (rev) mouse-specific quantitative PCR primers were as follows: GAPDH, 5′-AGCTTGTCATCAACGGGAAG-3′ (fwd) and 5′-TTTGATGTTAGTGGGGTCTCG-3′ (rev); GATA, 5′-CCAAGGCACGATCCAGCACAGA-3′ (fwd) and 5′-GGCCGACAGCCTTCGCTTGG-3′ (rev); IFNG, 5′-AGGTCAACAACCCACAGGTCC-3′ (fwd) and 5′-GATTCCGGCAACAGCTGGT-3′ (rev); IL-4, 5′-ACAGGAGAAGGGACGCCAT-3′ (fwd) and 5′-GAAGCCCTACAGACGAGCTCA-3′ (rev); IL-17A, 5′-AACTCCCTTGGCGCAAAAG-3′ (fwd) and 5′-GAGAGTCCAGGGTGACGTGG-3′ (rev); STAT4, 5′-CCTGGGTGGACCAATCTGAA-3′ (fwd) and 5′-CTCGCAGGATGTCAGCGAA-3′ (rev); STAT6, 5′-CCCCAACAAACTTCTCATCCA-3′ (fwd) and 5′-TTTGGCGTTGTTGTCTTGGTT-3′ (rev); and T-BET, 5′-ACCAACAACAAGGGGGCTCT-3′ (fwd) and 5′-CTCTGGCTCTCCATCATTCACC-3′ (rev).

### Quantification of *L. major* Parasites

To measure the parasite burden, genomic DNA was isolated using DNA purification solutions from QIAGEN (QIAGEN, Hilden, Germany). In brief, the cells were digested in cell lysis solution directly in the well. Protein was removed by adding protein precipitation buffer, and DNA was precipitated according to the manufacturer’s instructions with 100% 2-propanol (Sigma Aldrich, Taufkirchen, Germany). The concentration of mouse β-actin-DNA was quantified by PCR (65). The *Leishmania* DNA concentrations in the same samples were determined using fluorescence resonance energy transfer real-time PCR with Leishmanial 18S ribosomal DNA sequences (66). The resulting *Leishmania* DNA copy number was then divided by the copy number of β-actin-DNA to obtain the relative parasite units (RPU).

### Quantification of Cytokines

Supernatants of cell cultures were analyzed with a FlowCytomix™ kit (IL-13, IL-1α, IL-22, IL-2, IL-21, IL-6, IL-10, IL-27, IFN-γ, TNF-α, IL-4, and IL-17) according to the manufacturer’s (eBioscience, Thermo Fisher Scientific, Waltham, USA) specifications. Acquisition of probes was performed with a FACS Canto II (BD Bioscience).

### Statistical Analysis

Statistical significance between groups were calculated by different tests: two-way ANOVA for comparing the footpad swelling of analyzed groups, non-parametric Mann–Whitney tests if no normal distribution (no Gaussian distribution) was determined, and two-tailed Student’s *t*-test if normal distribution (Gaussian distribution) was determined. The calculation was performed using PRISM software (GraphPad, La Jolla, CA, USA). The corresponding *p* values are highlighted by stars only if *p* < 0.050.

## Results

### Local Infection with *Leishmania* Parasites Results in Systemic Expansion of Dectin-1^+^ DCs in Patients Suffering from CL

DCs are known to be important checkpoints for the generation of T cell-mediated protective immunity in experimental leishmaniasis (67). Based on the potential role of Dectin-1^+^ DCs in adaptive immunity, we addressed the question whether a cutaneous infection with *Leishmania* parasites results in an expansion of Dectin-1^+^ DCs.

**Figure 1:**
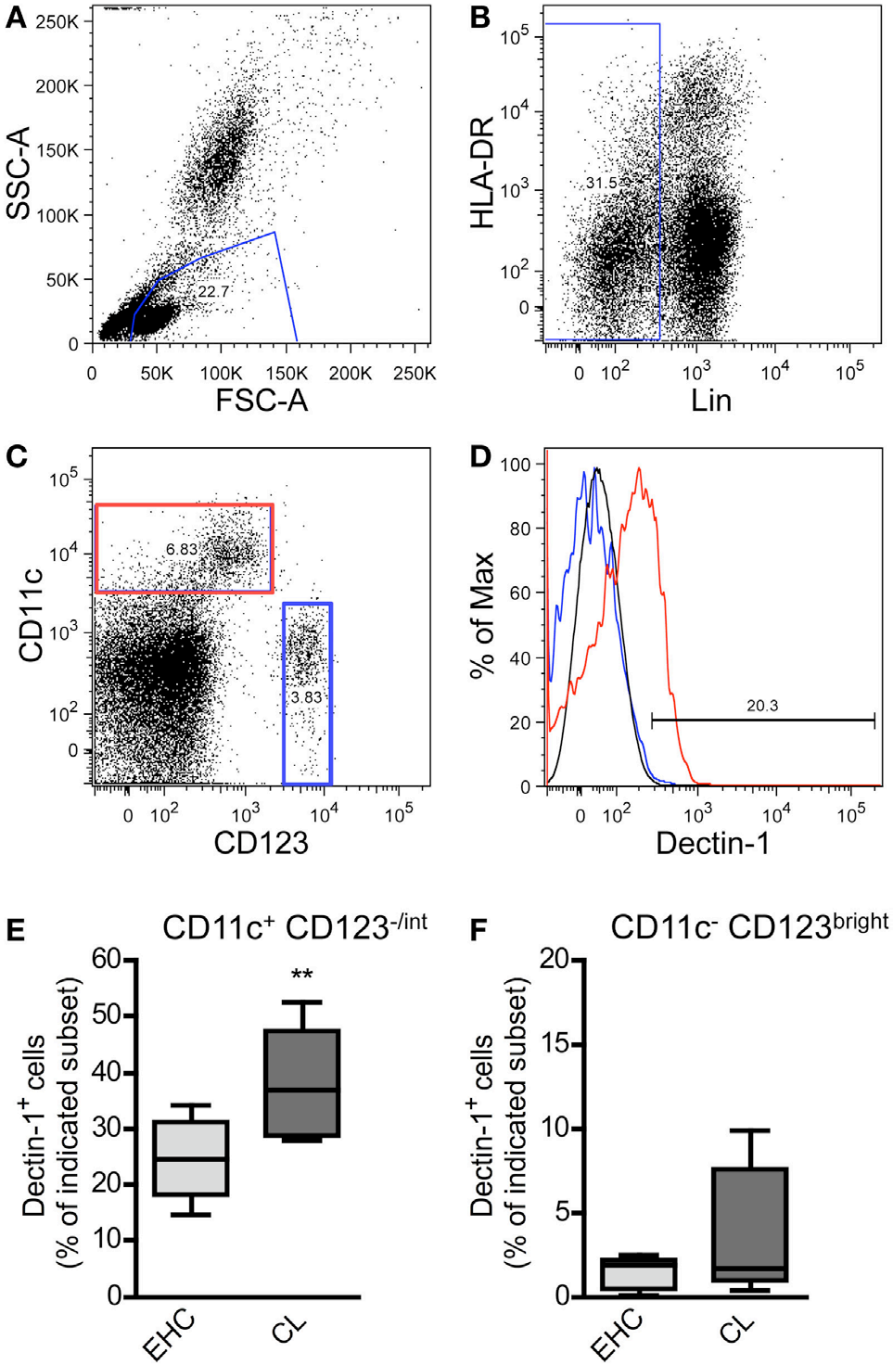
Characterization of Dectin-1^+^ DC subsets in blood samples from patients suffering from cutaneous leishmaniasis (CL). Peripheral blood samples from patients suffering from CL and Ethiopian healthy controls (EHCs) were stained for CD11c, Dectin-1, HLA-DR, CD123, and a lineage cocktail (anti-CD3, anti-CD14, anti-CD16, anti-CD19, anti-CD20, and anti-CD56) abbreviated as Lin. The following gating strategy was used. **(A)** FSC and SSC plots were used to define the leukocyte population. **(B)** Lin^−^ cells were selected for further analysis. **(C)** Lin^−^ cells were differentiated into CD11c^+^/CD123^−/int^ and CD11c^−^/CD123^bright^ populations displayed as dot plot. **(D)** The histogram plot indicates the expression of Dectin-1 on CD11c^+^/CD123^−/int^ (highlighted in red) and CD11c^−^/CD123^bright^ cells (highlighted in blue). The black line represents the isotype control. **(E,F)** The frequency of Dectin-1^+^ cells within the indicated subset is shown by box plot diagrams. Number of analyzed donors, *n* = 5. Data were analyzed using Student’s *t*-test (**p = 0.004).

In humans, it is difficult to analyze the dynamics of DC populations in tissues or secondary lymphoid organs. Therefore, we focused on the analyses of blood samples. DCs in the peripheral blood lack certain Lin-specific markers (68). Thus, Lin^−^ leukocytes were used for further characterization of human DCs (Figures 1A,B). The Lin^−^ population consists of HLA-DR^−^ and HAL-DR^+^ cells (Figure 1B). In accordance with other published data, the Lin^−^ CD11c^+^ cells are positive for HLA-DR and could be found within the Lin^−^/HLA-DR^+^ population (68, 69) (data not shown).

On the basis of the markers CD11c and CD123 (68, 70) myeloid DCs (mDC; CD11c^+^/CD123^−/int^) were dissected from plasmacytoid DC (pDC; CD11c^−^/CD123^bright^) subsets (Figure 1C). We could demonstrate that pDCs from EHCs and CL patients hardly express Dectin-1 (Figure 1D). The expression of Dectin-1 is predominantly restricted to mDCs (Figure 1D). Cutaneous infection with *Leishmania* parasites results in an increased frequency of Dectin-1^+^-positive mDCs in the periphery compared to healthy controls (Figure 1E). Of note, the pDC subset does not show significant differences in Dectin-1 expression between EHCs and patients suffering from CL (Figure 1F). These data suggest that a local infection with *Leishmania* parasite results in the expansion of Dectin-1^+^ mDC subsets in the peripheral blood of CL patients.

### Local Infection with *Leishmania* Parasites Results in Systemic Proliferation of Dectin-1^+^ DCs in BALB/c and C57BL/6 Mice

**Figure 2:**
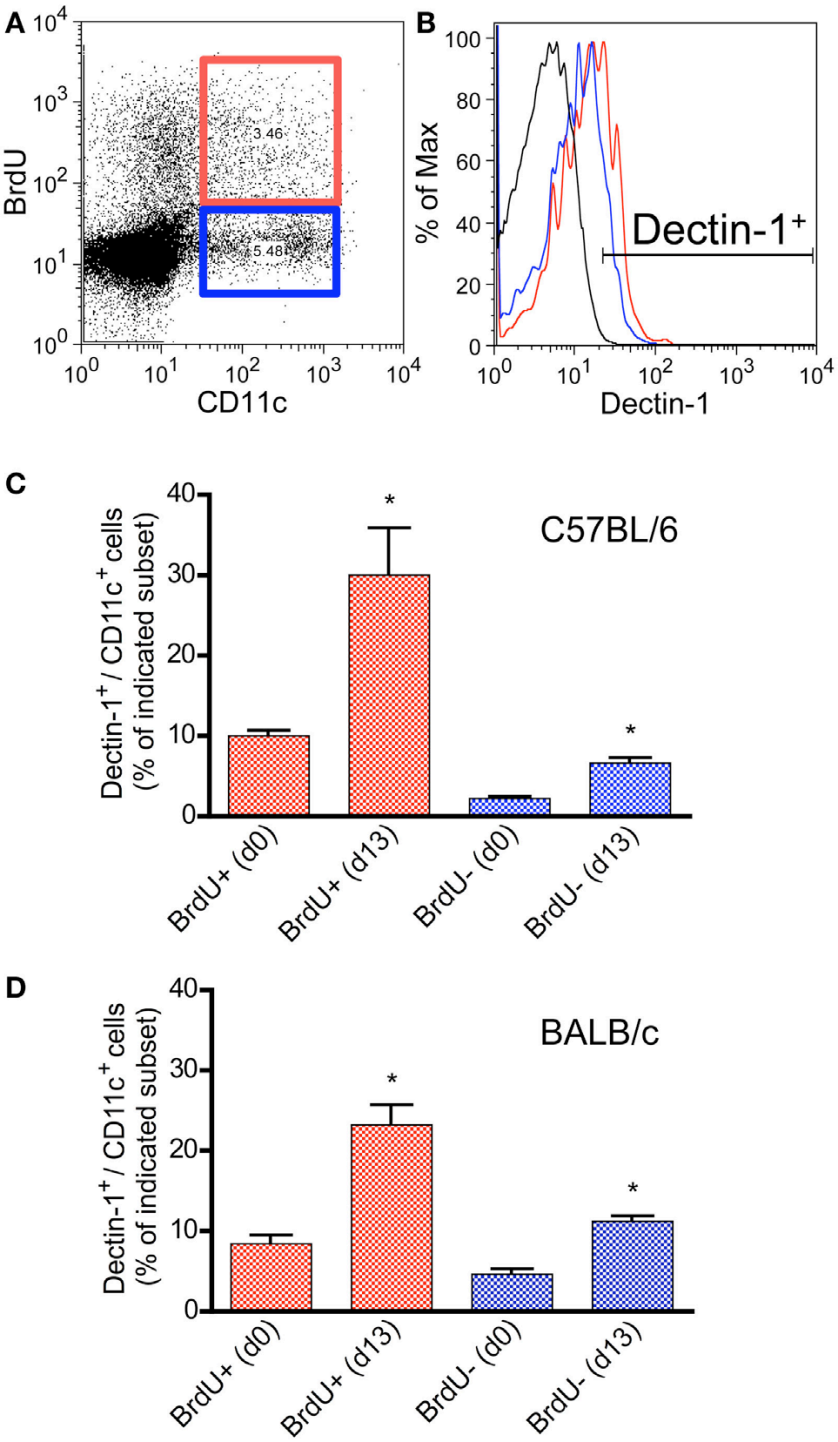
Characterization of Dectin-1 expression on proliferating CD11c^+^ DCs in blood samples of infected C57BL/6 and BALB/c mice. Mice were infected intradermally with *Leishmania major* parasites into the hind footpad, and the peripheral blood was analyzed 13 days after infection. Naïve mice served as controls (day 0). BrdU^+^ was given 3 days before the analysis. Peripheral blood leukocytes were gated. **(A)** The dot plot diagram displays the CD11c and BrdU intensities. BrdU^+^/CD11c^+^ cells are highlighted by a red and BrdU^−^/CD11c^+^ by a blue gate. **(B)** Dectin-1 expression of the BrdU subsets is shown as histogram displaying isotype control in black, BrdU^+^/CD11c^+^ cells in red, and BrdU^−^/CD11c^+^ cells in blue line. **(C)** The frequencies of Dectin-1^+^/CD11c^+^ cells (blue bars, BrdU^−^/CD11c^+^; red bars, BrdU^+^/CD11c^+^) within the peripheral blood of C57BL/6 mice were analyzed. Pooled data from three different experiments are shown (mean ± SD). Data were analyzed using Student’s *t*-test (**p = 0.004, ***p = 0.0003). **(D)** The frequencies of Dectin-1^+^/CD11c^+^ cells (blue bars, BrdU^−^/CD11c^+^; red bars, BrdU^+^/CD11c^+^) within the peripheral blood of BALB/c mice are indicated (mean ± SD). Pooled data from three different experiments were analyzed using the non-parametric Mann–Whitney test (*p < 0.5).

CD11c is hardly expressed by pDCs in mouse models (71, 72). Thus, most of the CD11c^+/bright^ DCs belong to the subset of conventional DCs including other mDC subsets (73, 74). In CL patients, Dectin-1^+^ mDCs expand after infection. To prove the concept that a local infection also results in a systemic expansion of Dectin-1^+^ mDCs, C57BL/6 and BALB/c mice were infected intradermally with *L. major* parasites. SDLNs, the site of infection, and the peripheral blood were analyzed for proliferating Dectin-1^+^ CD11c^+/bright^ DCs. Mice were administered the thymidine analog BrdU, as described in Section “Materials and Methods,” to analyze cell proliferation *in vivo*. 13 days after infection, when clinical symptoms such as footpad swelling occur and *Leishmania*-specific T cell-mediated immunity is initiated (13, 60), Dectin-1 was measured on BrdU^+^ (proliferating) and BrdU^−^ (resting) CD11c^+^ DCs (Figures 2A,B). Detailed quantification of Dectin-1^+^ DCs within proliferating and resting CD11c^+^ DCs revealed that both subsets express Dectin-1. However, the frequency of Dectin-1^+^ CD11c^+^ DCs is substantially higher within the proliferating subset compared to the resting CD11c^+^ DCs of BALB/c and C57BL/6 mice (Figures 2C,D). Further analysis of the site of infection and SDLNs of infected BALB/c and C57BL/6 mice confirmed this result (Figures S2A,B in Supplementary Material). These data generated from mice reflect the results obtained in CL patients, indicating that a local infection with *Leishmania* parasites results in a systemic expansion of Dectin-1^+^ DCs.

### The Presence of Curdlan at the Site of Infection Results in an Adaptive Immune Response in Normally Susceptible BALB/c Mice

**Figure 3:**
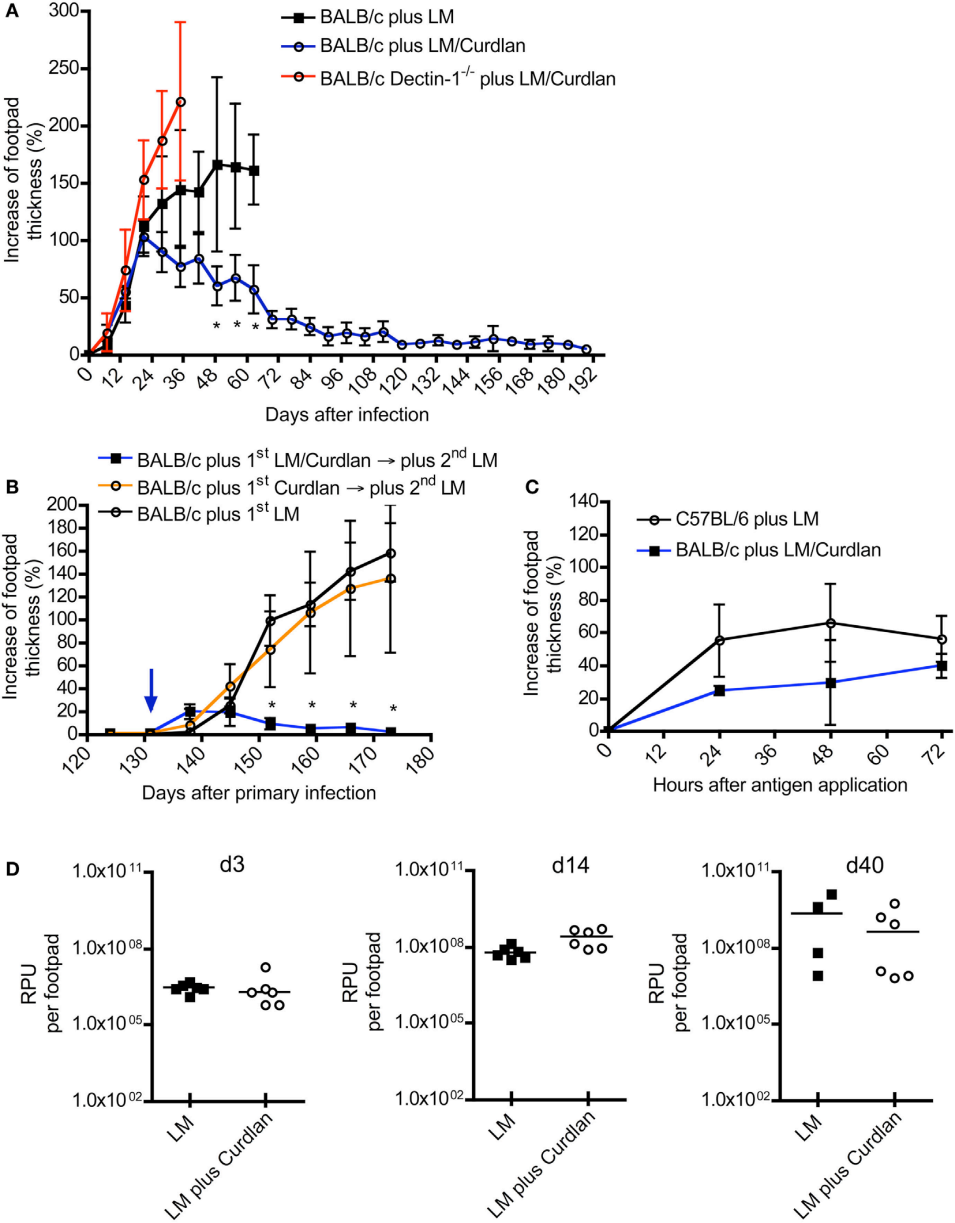
The presence of Curdlan at the site of infection results in an adaptive immune response in normally susceptible BALB/c. **(A)** BALB/c control mice were infected intradermally with *Leishmania (L.) major* into the right footpad (black line, black squares; 3 × 10^6^ parasites/30 μL; *n* = 19), abbreviated as BALB/c plus LM. Another group of BALB/c mice was infected with 30 µL of mixture of 3 × 10^6^ parasites and Curdlan (*c* = 50 μg/μL, blue line, black circles; *n* = 16), abbreviated as BALB/c plus LM/Curdlan. BALB/c mice deficient for Dectin-1 were also infected with 30 µL of a mixture of 3 × 10^6^ parasites and Curdlan (*c* = 50 µg/µL, red line, black circles; *n* = 5), abbreviated as BALB/c Dectin-1^−/−^ plus LM/Curdlan. Based on severe necrosis of the infected footpads, the groups BALB/c plus LM and BALB/c Dectin-1^−/−^ plus LM/Curdlan had to be terminated at days 60 and 36, respectively. The *y*-axis depicts the increase in footpad thickness (mean ± SD). Data were analyzed using Student’s *t*-test and highlighted if differences are significant (*p < 0.05; BALB/c plus LM compared to BALB/c plus LM/Curdlan). **(B)** Resistant BALB/c mice that had been treated with Curdlan in the presence of *L. major* parasites were reinfected at day 131 (blue arrow) after primary infection (abbreviated as BALB/c plus 1st LM/Curdlan → 2nd LM, blue line, black squares; *n* = 3). BALB/c mice that had been treated with Curdlan alone 131 days before (abbreviated as BALB/c plus 1st Curdlan → 2nd LM, orange line, black circles; *n* = 3) were also infected. Naïve BALB/c mice were infected (BALB/c plus 1st LM, black line, black circle; *n* = 5), too. The *y*-axis depicts the increase in footpad thickness (mean ± SD). Data (BALB/c plus 1st LM compared to BALB/c plus 1st LM/Curdlan → 2nd LM) were analyzed using two-way ANOVA (*p < 0.05). **(C)** A delayed-type hypersensitivity (DTH) response is shown, comparing C57BL/6 mice infected with 3 × 10^6^ parasites (plus LM, black line, black circles; *n* = 3) with BALB/c mice infected with 3 × 10^6^ parasites plus Curdlan (plus LM/Curdlan, blue line, black squares; *n* = 3). The DTH response was induced 217 days after primary infection as described in Section “Materials and Methods.” The *y*-axis depicts the increase in footpad thickness (mean ± SD), the *x*-axis hours after antigen application. **(D)** Relative parasite units (RPU) representing the relative amount of parasites per footpad at days 3, 14, and 40 after infection are displayed. Infected BALB/c mice (LM, black squares) were compared with BALB/c mice that had been infected with parasites plus Curdlan (LM plus Curdlan, black circles). The mean (horizontal line) is shown. Each circle or square represents one analyzed footpad. Data were analyzed using Student’s *t*-test (d3 *p* = 0.779, d14 *p* = 0.062, and d40 *p* = 0.286).

So far, only systemically (i.p. and/or i.v.) applied β-glucans have been used to treat BALB/c or C57BL/6 mice after infection (34, 43–48). No published data exist analyzing the effects of cutaneous application of β-glucan such as Curdlan in parallel to *L. major* infection. Given that Dectin-1^+^ DCs expand at the site of infection and within SDLNs (Figure 2; Figure S2 in Supplementary Material), it seems feasible that a local stimulation of Dectin-1^+^ DCs with Curdlan modulates the parasite-specific immune response. To test this hypothesis, BALB/c and C57BL/6 mice were infected with promastigote parasites suspended in PBS plus Curdlan. The course of infection was not substantially modulated in resistant C57BL/6 mice (Figure S3 in Supplementary Material). In contrast, normally susceptible BALB/c mice showed a resistant phenotype when the parasites were intradermally injected in combination with Curdlan (Figure 3A). BALB/c mice infected with *L. major* alone displayed the well-known severe course of disease (Figure 3A). An additional control experiment with Dectin-1^−/−^ BALB/c was performed to confirm the specificity of the Curdlan/Dectin-1 interaction *in vivo*. Here, we were able to demonstrate that an application of Curdlan together with the parasites does not result in protective immunity against *L. major* parasites as shown in protected BALB/c wild-type mice (WT) (Figure 3A; Figure S4 in Supplementary Material). The experiments with non-healing BALB/c WT and Dectin-1^−/−^ mice had to be terminated based on the severe ulcerated footpads.

C57BL/6 mice usually resolve a primary infection with *L. major*. Following resolution of the primary infection, they are immune to reinfection due to the generation of antigen-specific memory T cells (75, 76). To test whether Curdlan-protected BALB/c mice (Figure 3A) show classical signs of adaptive immunity, reinfection experiments were performed. BALB/c mice that had received Curdlan intradermally in the absence of parasites were used to exclude the possibility of an unspecific expansion of T cells potentially mediating protection. Resistant BALB/c mice, that had been infected with *L. major* parasites in the presence of Curdlan, were reinfected into the contra lateral footpad at day 131 after primary infection with a high-dose of parasites in the absence of Curdlan. At this time point, no clinical signs such as footpad swelling were detectable. BALB/c mice that have been treated intradermally only with Curdlan 131 days before the primary infection developed a severe course of disease, that is, comparable to infected BALB/c mice that were not pretreated (Figure 3B). In contrast, protected BALB/c mice, that resolved the primary infection, were able to rapidly control the second round of a high-dose infection with *Leishmania* parasites in the absence of Curdlan (Figure 3B). These mice now show symptoms of an infection until the termination of the experiment 120 after the second exposition to *Leishmania* parasites in the absence of Curdlan.

Beside a reinfection, the DTH response is also an accepted indicator for the presence of an adaptive T cell-mediated immune response (75, 77). In contrast to resistant C57BL/6 mice, BALB/c mice hardly generate a measurable DTH reaction after a high-dose infection with *L. major* parasites (52). Using the high-dose model, we were able to demonstrate that Curdlan-protected BALB/c mice can indeed develop a DTH reaction that is comparable to that of resistant C57BL/6 mice (Figure 3C). Consequently, protected BALB/c mice mount an adaptive T cell-mediated immunity against *L. major* parasites that is accompanied by a clear reduction of the parasite load at the site of infection during the effector phase of experimental leishmaniasis (Figure S5 in Supplementary Material).

It needs to be mentioned that BALB/c mice can develop protective immunity against *L. major* if they have been infected with a low dose of parasites (8). Thus, we had to exclude that skin-homing macrophages eliminate the parasites after Curdlan contact shortly after infection. A milder course of disease would be the artificial consequence. In line with published data (31, 33), we were able to demonstrate that macrophages express Dectin-1 in the presence or absence of parasites (Figures S6A–D in Supplementary Material). However, Curdlan activation does not induce leishmanicidal mechanisms resulting in the elimination of *L. major* parasites (Figure S7 in Supplementary Material). In line with this finding, we were able to show that Curdlan-treated and control BALB/c mice show the same parasite burden at the site of infection during the early phase (days 3 and 14) of experimental leishmaniasis (Figure 3D). At day 40, after infection the beneficial effect of Curdlan treatment (the effect of Curdlan treatment on the parasite load at day 80 after infection, is shown in Figure S5 in Supplementary Material) is slightly indicated but not significant (Figure 3D).

In conclusion, we can demonstrate that the presence of Curdlan at the time of intradermal infection does not induce antigen-unspecific side effects but triggers the modulation of an adaptive T-cell response of BALB/c mice in a Dectin-1-dependent manner: they develop a resistant phenotype.

### Cutaneous Curdlan Application Dampens the Th2 Response of Infected BALB/c Mice

The rapid clearance of *L. major* parasites after a high-dose reinfection and the positive DTH response of protected BALB/c mice (Figures 3B,C) supports the hypothesis that an adaptive Th1 response was generated. To assess that possibility, *Leishmania*-specific IgG subtypes, Th1- and Th2-indicating cytokines, and selected transcription factors were characterized.

**Figure 4:**
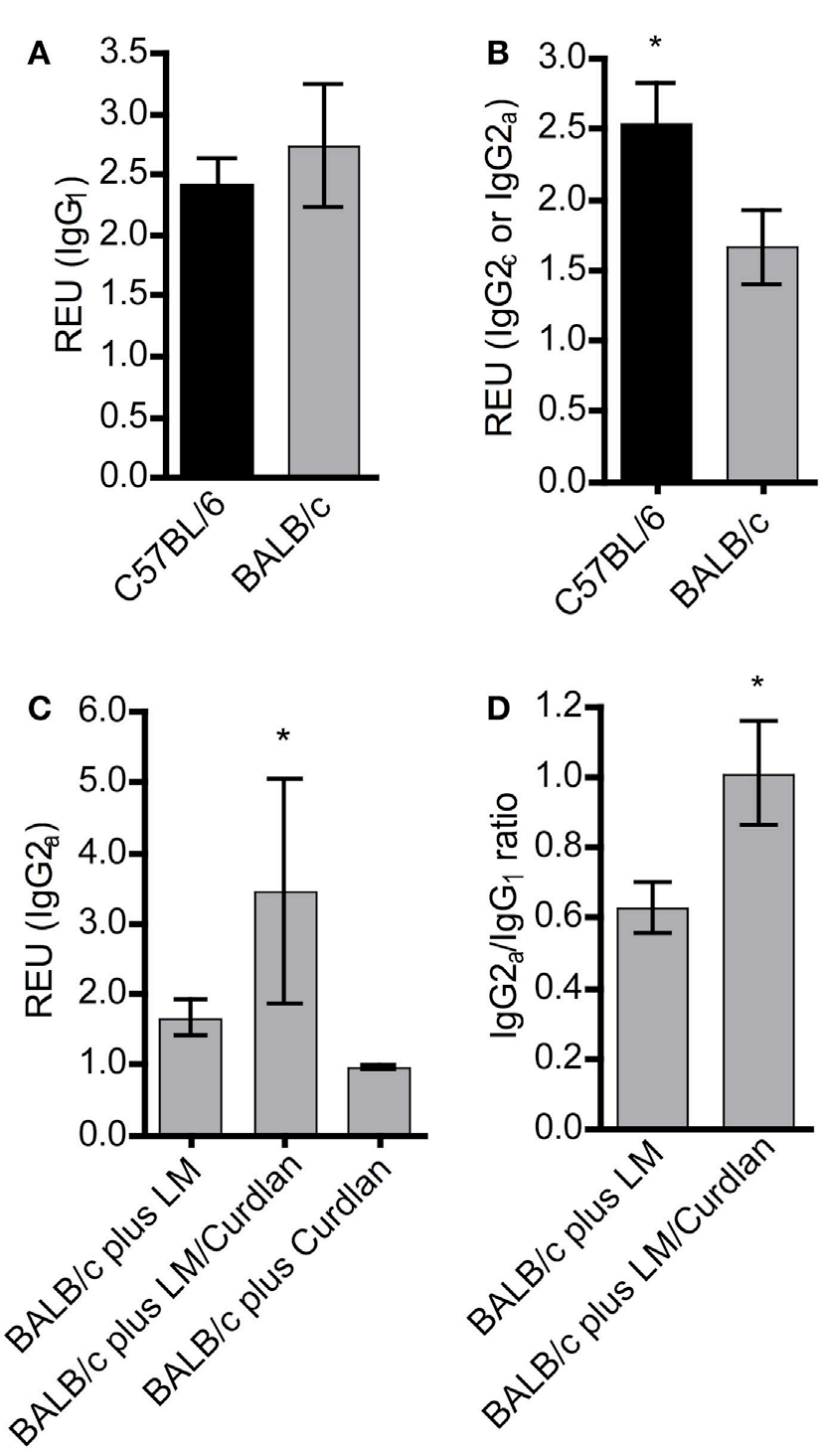
The presence of Curdlan at the site of infection modulates the humoral immune response of BALB/c mice toward a pronounced production of *Leishmania*-specific IgG2a. C57BL/6 and BALB/c mice were infected with a volume of 30 µL PBS containing 3 × 10^6^ *Leishmania (L.) major* (LM) parasites in the presence or absence of Curdlan (*c* = 50 µg/µL). Blood samples were collected 25 days after infection. *Leishmania*-specific IgG_1_, IgG_2a_, and IgG_2c_ in sera of infected mice were quantified by ELISA as described in Section “Materials and Methods.” **(A)** Relative ELISA units (REU) of *Leishmania*-specific IgG_1_ of infected BALB/c and C57BL/6 mice are depicted (*n* = 3). **(B)** REU of *Leishmania*-specific IgG_2a_ and IgG_2c_ of infected BALB/c and C57BL/6 mice were compared (*n* = 3; *p < 0.05). **(C)** REU of *Leishmania*-specific IgG_2a_ of three groups are shown: infected BALB/c mice (plus LM), infected BALB/c mice in the presence of Curdlan (plus LM/Curdlan), and Curdlan-treated BALB/c mice (plus Curdlan) (*n* = 3; *p = 0.043). **(D)** The ratio of *Leishmania*-specific IgG_2a_/IgG_1_ was calculated in BALB/c mice infected with *L. major* (plus LM) and BALB/c mice infected with *L. major* in the presence of Curdlan (plus LM/Curdlan) (*n* = 3; *p < 0.05). Data were analyzed using the non-parametric Mann–Whitney test. The mean ± SD is displayed.

Protective immunity to *L. major* infection does not depend on the production of specific Abs (78). However, *L. major*-specific IgG responses are an indication for the ability of the host organism to mount an antigen-specific immune reaction. In experimental leishmaniasis, BALB/c mice mount a Th2 response and produce predominantly IgG_1_, whereas resistant C57BL/6 mice develop a Th1 response and an increased production of IgG_2c_ Abs (3, 79). An increase in parasite-specific IgG_2c_ is therefore an indicator for an ongoing protective Th1 response. In mouse strains with the *Igh1-a* allele, like BALB/c, the IgG_2c_ gene is deleted (80). Thus, we determined *Leishmania*-specific IgG_1_ and IgG_2a_ subsets in sera of BALB/c mice that had been infected in the presence or absence of Curdlan. First, we analyzed mice that had not been treated with Curdlan. C57BL/6 and BALB/c mice generated *Leishmania*-specific IgG_1_ 25 days after infection (Figure 4A). In contrast, C57BL/6 mice produced a significantly higher amount of IgG_2a_ isotypes compared to susceptible BALB/c mice (Figure 4B). The infection in the presence of Curdlan led to an increase in *Leishmania*-specific IgG_2a_ in BALB/c mice (Figure 4C) that also provoked a higher IgG_2a_/IgG_1_ ratio of *Leishmania*-specific Abs (Figure 4D). Based on these data, we conclude that Curdlan treatment might dampen the development of a disease-promoting Th2 response in BALB/c mice.

In our study, we used a high-dose infection model and could demonstrate that Curdlan application results in protective immunity in BALB/c mice (Figure 3). It is important to mention that BALB/c mice do not necessarily develop a severe course of disease. In low-dose infection models, BALB/c mice develop a resistant phenotype, accompanied by the polarization toward a Th1-like phenotype (8). Thus, CD4^+^ T cells from BALB/c mice can also differentiate into Th1 cells.

To address the question, whether protected BALB/c mice show a Th1 or Th2 profile, CD4^+^ T cells were purified from SDLNs and analyzed by qRT-PCR. It is commonly accepted that activated CD4^+^ T cells that receive IL-4 signaling upregulate GATA-binding protein 3 (GATA3) and become capable of producing Th2 cytokines (81, 82). Thus, GATA3 is regarded as an inducer for Th2-cell differentiation (83).

We measured the relative expression of GATA3 in CD4^+^ T cells isolated from SDLNs of infected BALB/c mice. These qRT-PCR data revealed that Curdlan treatment results in a dampened expression of GATA3 at day 10 (Figure S8A in Supplementary Material), which is significantly reduced at day 28 (Figure S8C in Supplementary Material) after infection compared to control animals. Furthermore, four out of five Curdlan-treated BALB/c mice also showed a clear reduction in IL-4 expression within the pool of CD4^+^ T cells (Figures S8B,D in Supplementary Material). Thus, a cutaneous application of Curdlan seems to dampen an early IL-4 production within SDLNs. Surprisingly, CD4^+^ T cells isolated from SDLN of Curdlan-treated mice showed a transient trend toward a lower expression of the key transcription factor T-bet, known to define Th1 cells (84), and of the Th1 cytokine IFN-γ at day 10 after infection (Figures S8E,F in Supplementary Material). In contrast, at day 28 the expression of T-bet (Figure S8 in Supplementary Material) but not of IFN-γ (Figure S8H in Supplementary Material) was slightly increased in CD4^+^ T cells of Curdlan-treated BALB/c mice.

**Figure 5:**
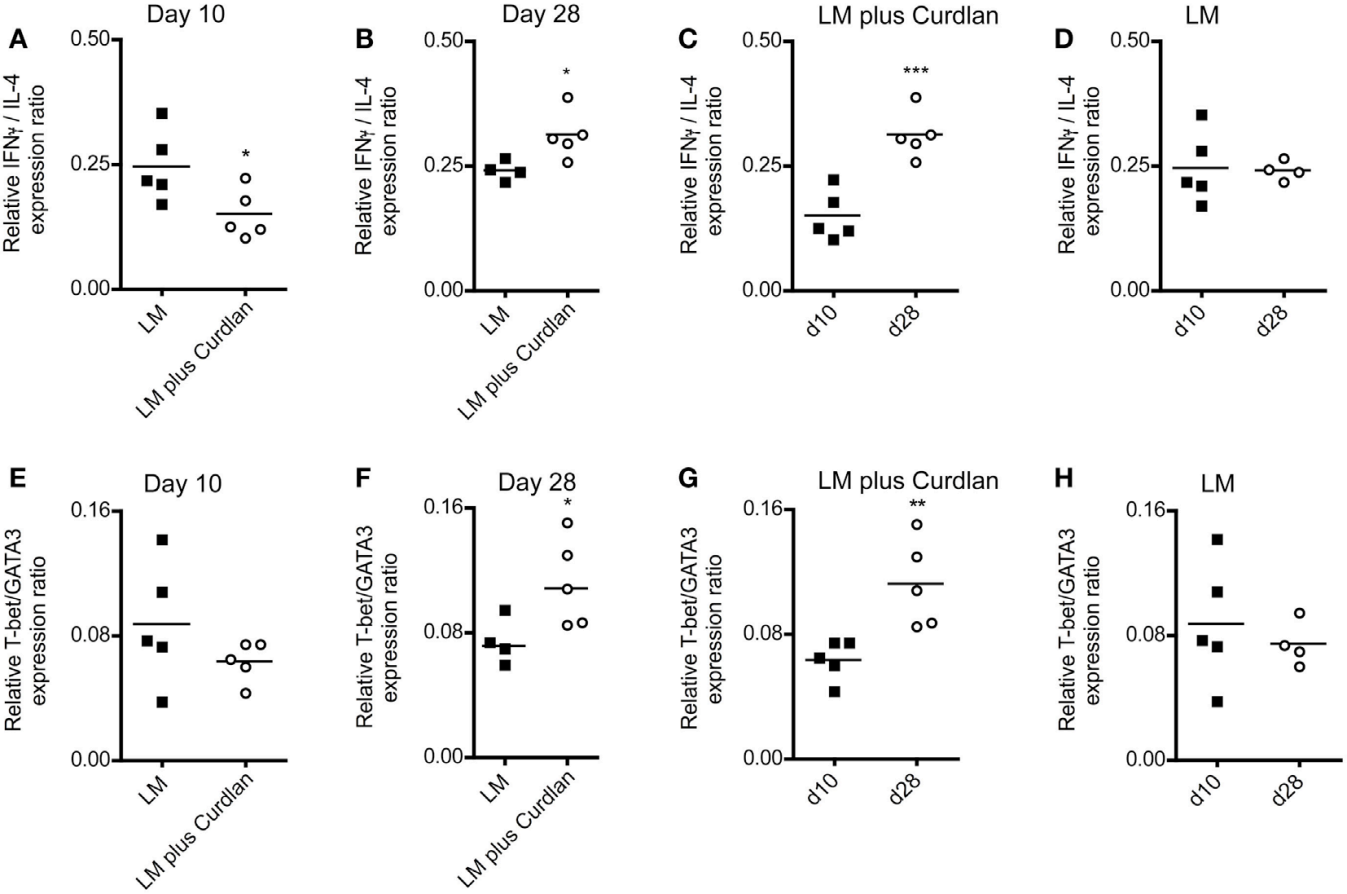
Lymph node-resident CD4^+^ T cells from Curdlan-treated BALB/c mice show a shift toward an impaired T helper (Th) 2 response. BALB/c mice were infected with a volume of 30 µL PBS containing 3 × 10^6^ *Leishmania (L.) major* (LM*)* parasites in the presence (abbreviated as LM plus Curdlan, *c* = 50 µg/µL) or absence of Curdlan (abbreviated as LM). CD4^+^ T cells were purified at days 10 and 28 after infection, and qRT-PCR was performed to determine the relative mRNA levels of target genes referred to GAPDH. The ratios of T-bet/GATA-binding protein 3 (GATA3) and of IFN-γ/IL-4 relative mRNA levels are shown. **(A,B)** The scatter plots depict the relative expression ratio of IFN-γ/IL-4 from the analyzed groups (LM and LM plus Curdlan) at day 10 [**(A)**, *p = 0.042] and day 28 [**(B)**, *p = 0.032]. **(C,D)** The scatter plots depict the relative expression ratios of IFN-γ/IL-4 from the analyzed groups in a time-dependent manner. **(C)** LM plus Curdlan (***p = 0.0007) and **(D)** plus LM (*p* > 0.05). **(E,F)** The scatter plots depict the relative expression ratio of T-bet/GATA3 from the analyzed groups (LM and LM plus Curdlan). Day 10 [**(E)**, *p* > 0.05] and day 28 [**(F)**, *p = 0.048] are shown. **(G,H)** The scatter plots depict the relative expression ratios of T-bet/GATA3 from the analyzed groups in a time-dependent manner. **(G)** LM plus Curdlan (**p = 0.008) and **(D)** plus LM. Data were analyzed using Student’s *t*-test. Each symbol represents an individual mouse, and the bars indicate the medians.

A rich body of literature described the fact that Th-cell responses are characterized by the balance of Th1- and Th2-associated cytokines and not by the relative amount of single cytokines such as IFN-γ and IL-4 (85–90). Consequently, we calculated the Th1/Th2 ratio based on the relative expression levels of GATA3, IL-4, T-bet, and IFN-γ at days 10 and 28 after infection. As shown in Figures 5A,B, cutaneous Curdlan application was associated with a modulation of the IFN-/IL-4 ratios at day 10 and 28, indicating a shift toward a pronounced Th1 and a dampened Th2 response at day 28 after infection (Figure 5C). The IFN-/IL-4 ratios of BALB/c mice, infected in the absence of Curdlan showed no modification (Figure 5D). In line with this findings, the ratios of Th1-, Th2-driving transcription factors (T-bet/GATA3) was not substantial different at day 10 but significant enhanced at day 28 (Figures 5E,F), if mice had been treated with Curdlan. Additionally, we were able to demonstrate that this Curdlan-induced Th1 polarization increased from day 10 to day 28 after infection (Figure 5G). T-bet/GATA3ratios of BALB/c mice, infected in the absence of Curdlan showed no modification (Figure 5H). The Th1 polarization was also accompanied by a pronounced IL-17A mRNA expression of CD4^+^ T cells (data not shown).

### *L. major-*Harboring DCs Express Higher Levels of Dectin-1 and Start to Mature after Curdlan Stimulation

As shown earlier, Dectin-1^+^ DCs expand at the site of infection and within SDLNs after infection with *Leishmania* parasites (Figure 2). A dermal exposure to Curdlan can protect BALB/c mice from severe leishmaniasis and induces T cell-mediated immune responses protecting from a high-dose reinfection (Figure 3). Cutaneous DCs are known to be crucial for the generation of adaptive immunity. These professional APCs capture parasites or parasite-derived antigens and are able to migrate to SDLNs for subsequent antigen presentation to T cells (14). To accomplish these tasks, DCs undergo a maturation program accompanied by the expression of surface molecules pivotal for migration and of costimulatory molecules (17). Thus, we wanted to find out whether Dectin-1^+^ DCs get infected by parasites and if these parasite-harboring Dectin-1^+^ DCs undergo maturation after Curdlan treatment.

**Figure 6:**
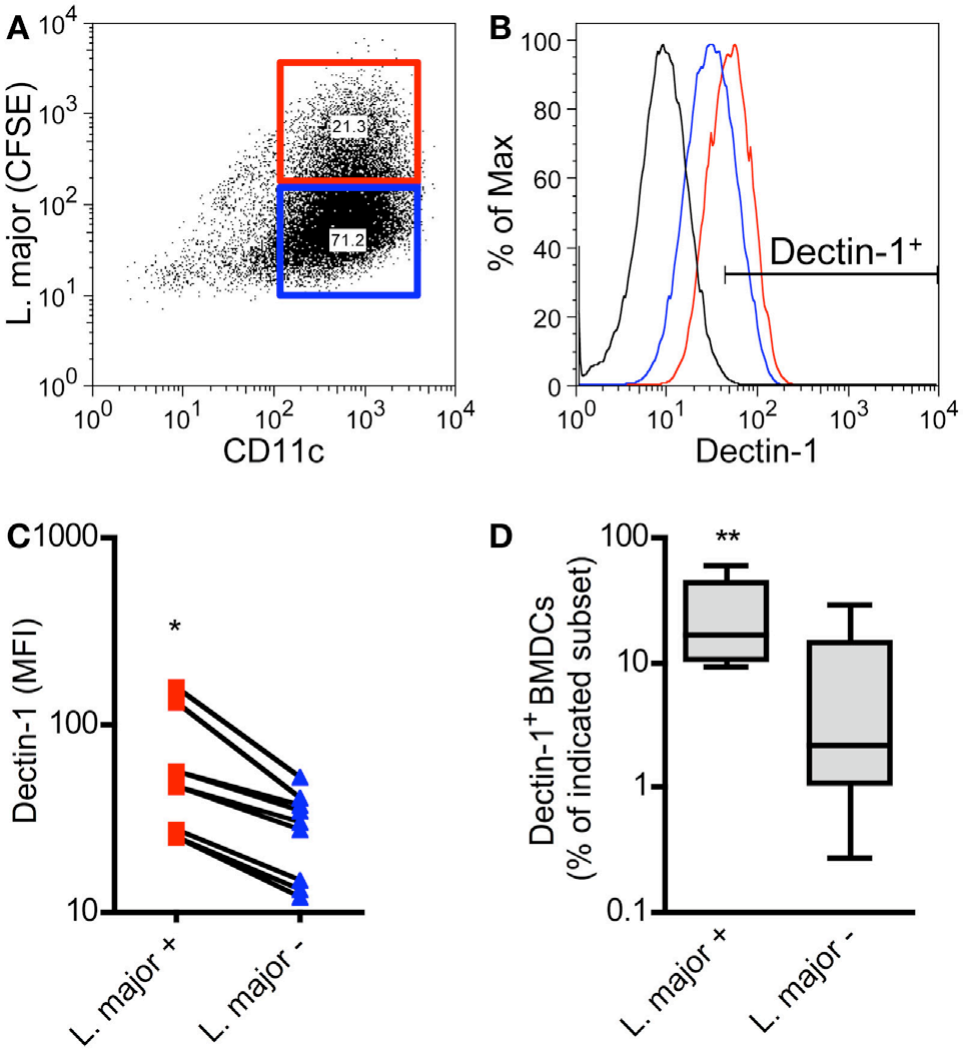
Dectin-1 is expressed to a stronger degree by *Leishmania (L.) major* harboring bone marrow-derived dendritic cells (BMDCs) compared to non-infected BMDCs. BMDCs were infected with CFSE-labeled *L. major* promastigote parasites. After 24 h, BMDCs were harvested and characterized by flow cytometry analysis for the markers CD11c and Dectin-1. **(A)** The dot plot diagram depicts two populations of *L. major* harboring (*L. major* CFSE^+^; red square) and not infected (*L. major* CFSE^−^; blue square) CD11c^+^ BMDCs. The gates were placed according to the isotype controls of not infected BMDCs. **(B)** The histogram visualizes the expression of Dectin-1 of infected (*L. major*^+^; red line) and not infected (*L. major*^−^; blue line) BMDCs. The black line depicts the isotype control. **(C)** The mean fluorescence intensity (MFI) of Dectin-1 expressed by *L. major*^+^ (red squares) and not infected subsets (*L. major*^−^; blue triangles) is shown. Each symbol represents an individual mouse. Pooled data of three independent experiments are included (*p = 0.018). **(D)** The frequency of Dectin-1^+^ cells within the infected (*L. major*^+^) and not infected (*L. major*^−^) subsets is displayed as a box plot (*n* = 5 experiments, **p = 0.0029). Data were analyzed using the non-parametric Mann–Whitney test.

BMDCs were cocultured with CFSE-labeled promastigote parasites. This allows the differentiation of infected versus non-infected BMDCs subsets simultaneously (Figure 6A) (57). With this method, we could demonstrate that *L. major^+^* BMDCs express a higher level of Dectin-1 compared to uninfected DCs (Figures 6B,C). The frequency of Dectin-1-positive BMDCs was also significantly higher in the infected BMDC population compared to BMDCs that were negative for *L. major* parasites (Figure 6D). Comparable to BMDCs, BMDMs show also a moderate increase in the frequency of Dectin-1^+^ cells and Dectin-1 expression intensity after exposition to *L. major* parasites (Figure S6 in Supplementary Material). This would suggest Dectin-1-mediated host pathogen interactions. Thus, we addressed the question whether pathogen-derived carbohydrates can interact with C-type lectins and especially with Dectin-1.

The CLRs DC-SIGN (CD209) and the mannose receptor C-type 1 (CD206) (91) have been shown to bind *Leishmania* antigens. However, it is unknown whether Dectin-1 recognizes *L. major-*derived molecules. With the help of selected CLR-Fc fusion proteins, we were able to demonstrate an interaction of *L. major* parasites with CLEC-9 and MGL-1 that recognizes f-actin (92) or galactose, respectively (93) (Figure S9 in Supplementary Material). However, a binding of Dectin-1-Fc fusion proteins to parasite structures could not be observed (Figure S9 in Supplementary Material). Consequently, even though myeloid cells such as macrophages and DCs are positive for Dectin-1 and show a higher Dectin-1 expression after parasite exposition, Dectin-1 alone is not crucial for the uptake of *L. major* parasites, because the parasites are negative for the Dectin-1 ligand. The fact that Dectin-1^−/−^ phagocytes can still take up parasites supports that conclusion (31).

**Figure 7:**
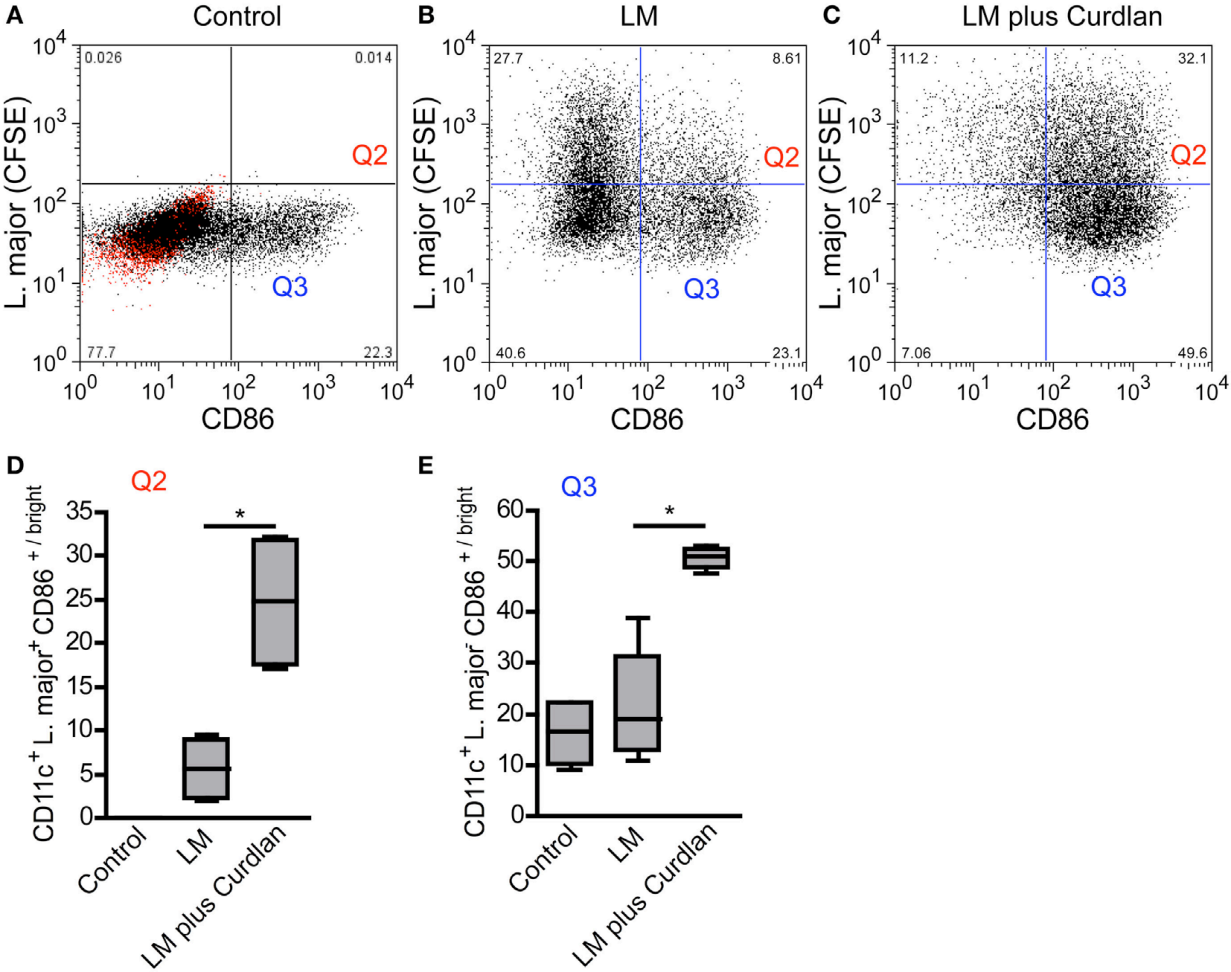
*Leishmania (L.) major* (LM) harboring bone marrow-derived dendritic cells (BMDCs) mature after Curdlan stimulation. The effect of Curdlan on BMDC maturation was analyzed. 24 h after the Curdlan stimulation, BMDCs were harvested and characterized by flow cytometry using the markers CD11c and CD86. **(A)** The dot plot diagram depicts CD11c^+^ BMDCs that had been cultured in the absence of Curdlan and CFSE-labeled *L. major* promastigote (control). The red population indicates the isotype control for the anti-CD86 antibody. The quadrants of interest are upper right (Q2, red; *L. major* CFSE^+^/CD86^+/bright^) and lower right (Q3, blue: *L. major* CFSE^−^/CD86^+/bright^). The quadrants were placed according to the isotype control and CD11c^+^ BMDCs negative for CFSE-labeled parasites. **(B)** The dot plot diagram depicts the quadrants of interest of CD11c^+^ BMDCs incubated with CFSE-labeled parasites (LM). **(C)** The dot plot diagram depicts the quadrants of interest of Curdlan stimulated CD11c^+^ BMDCs cocultured with CFSE-labeled parasites (LM plus Curdlan). **(D)** The frequency of *L. major* CFSE^+^ and CD86^+/bright^ BMDCs (Q2) is shown. **(E)** The frequency of *L. major* CFSE^−^ and CD86^+/bright^ (Q3) BMDCs is depicted. The following culture conditions were compared: not infected, not stimulated BMDCs [Control, compare **(A)**], *L. major* harboring BMDCs [LM, compare **(B)]**, and *L. major* harboring BMDCs stimulated with Curdlan [LM plus Curdlan, compare **(C)**]. The box plots **(D,E)** depict pooled data out of two independent experiments (*n* = 4). Data were analyzed using the non-parametric Mann–Whitney test [**(C,D)**, *p = 0.0286].

*L. major* parasites can be detected preferentially in BMDCs showing an immature phenotype (Figures 7A,B) and do not induce a maturation of infected cells (data not shown). Thus, parasites seem to inhibit the maturation of immature and Dectin-1^high^ DCs and thereby the subsequent migration to the SDLN (94, 95). The incubation of infected BMDCs with Curdlan is accompanied by a strong release of TNF-α and IL-6 and a maturation of infected BMDCs (Figure 7C; Figure S10 in Supplementary Material). This is of special interest, because TNF-α and IL-6 are known to be crucial for DC maturation and subsequent priming of T cells (17, 18). Based on the cytokine microenvironment, infected and non-infected Curdlan-treated BMDCs express high levels of CD86 and thus present a mature phenotype (Figures 7D,E). The stimulation of infected BMDCs with Curdlan also induces a strong release of T-cell growth factor IL-2 (Figure S10 in Supplementary Material). In association with the massive induction of costimulatory molecules such as CD86, Curdlan seems to represent an ideal adjuvant for efficient priming of *L. major-*specific T cells (96). In line with other published data, we showed that Curdlan activates BMDC maturation in a Dectin-1-specific manner (Figure S11 in Supplementary Material) (97, 98).

Additionally, we addressed the question whether Dectin-1 can be detected on Curdlan-activated BMDCs. In this context, we were able to demonstrate that immature BMDCs show in general a higher Dectin-1 expression compared to mature BMDCs (Figure S12A in Supplementary Material), and the detection of surface Dectin-1 is diminished after Curdlan stimulation (Figures S12B,E in Supplementary Material). The population of mature BMDCs, that is positive for *L. major* parasites, shows also a reduction in the Dectin-1 expression after stimulation with Curdlan (compare Q2, Figure S13 in Supplementary Material). BMDCs that have been exposed to parasites but do not harbor parasites, show no substantial reduction in Dectin-1 expression (compare Q2, Figure S13 in Supplementary Material).

In conclusion, we were able to demonstrate that Curdlan activation results in a maturation of infected BMDCs in a Dectin-1-specific manner that in turn might be crucial for the efficient priming of antigen-specific CD4^+^ T cells.

### Curdlan-Activated DCs Show a Pronounced Potential of Expanding *Leishmania*-Specific CD4^+^ T Cells

**Figure 8:**
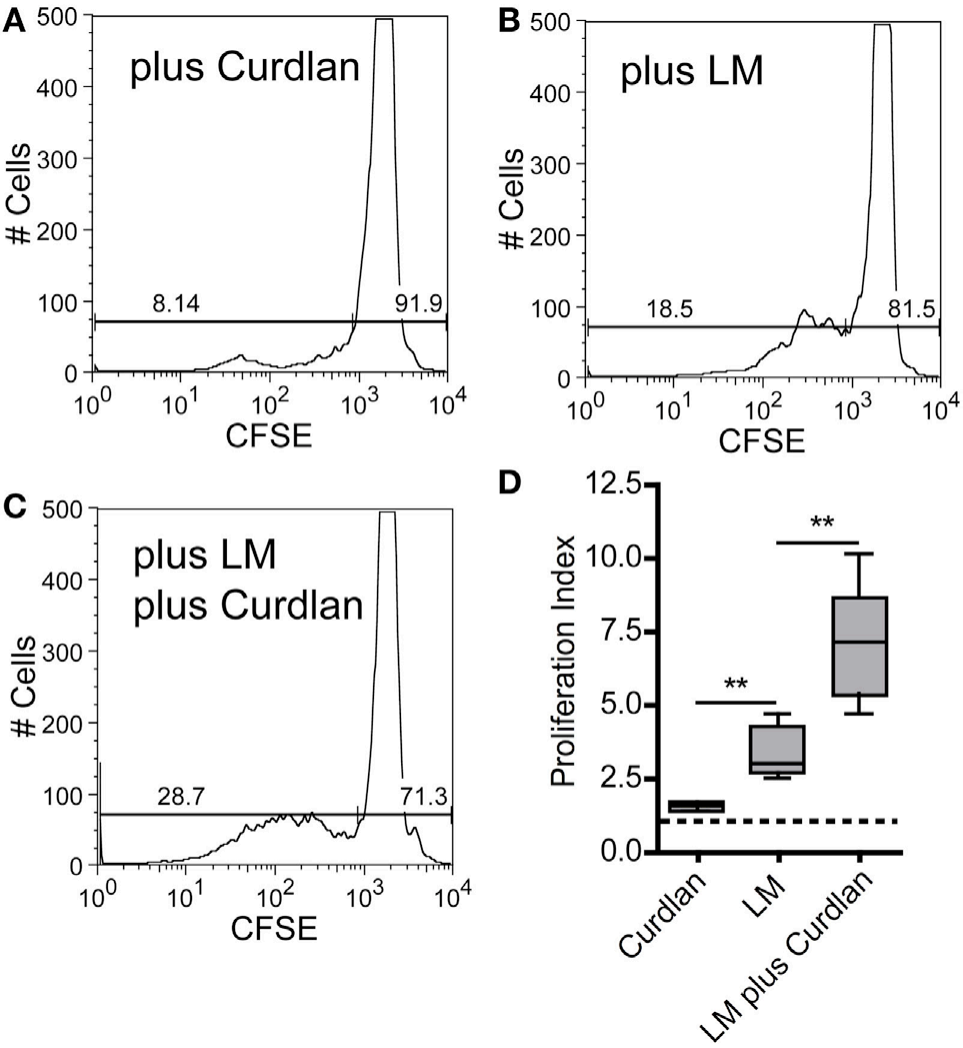
Curdlan stimulation enhances the potential of parasite harboring bone marrow-derived dendritic cells (BMDCs) toward the expansion of *Leishmania*-specific CD4^+^ T cells. BALB/c mice were infected with a volume of 30 µL PBS containing 3 × 10^6^ *Leishmania (L.) major* parasites. Ten days after infection, CD3^+^ T cells were isolated from skin-draining lymph nodes (SDLNs) and labeled with CFSE. BALB/c BMDCs were harvested at day 10 after *ex vivo* differentiation with GM-CSF and incubated for 24 h with *L. major* parasites (5:1) in the presence or absence of Curdlan (50 μg/200 μL). CFSE-labeled CD3^+^ T cells and BMDCs (10:1) were incubated for 72 h and analyzed by flow cytometry. The proliferative (left gate) and resting (right gate) CD4^+^ T cells are visualized by gates within the histogram plots. Following experimental approaches are displayed: **(A)** Curdlan activated but not infected BMDCs (plus Curdlan), **(B)** infected but not activated BMDCs (plus *L. major*), and **(C)** Curdlan-activated and infected BMDCs (plus *L. major;* plus Curdlan). The percentage of resting and proliferating cells is indicated inside the corresponding gates. **(D)** The proliferation index (described in detail in Section “Materials and Methods”) is displayed as a box plot diagram. Pooled data from two independent experiments are shown. Data were analyzed using Student’s *t*-test (Curdlan compared with LM, **p = 0.0018 and LM plus Curdlan compared with LM, **p = 0.0058; *n* = 5).

The adjuvant effect of Dectin-1 ligation in T-cell immunity is undisputed, and it has been proven that Curdlan can polarize T-cell responses (99, 100). In line with these data, it has been shown that anti-Dectin-1 stimulation and the application of microparticulate β-glucan conjugates can potentiate an ovalbumin-specific CD4^+^ T-cell response (101, 102). It was also described that Curdlan treatment can switch a Th2 to a Th1 response in tumor immunology (103). Last but not least, Dectin-1 ligation is discussed to be an important checkpoint in Th17-mediated immunity (28, 34, 38, 97, 104). However, the potential of Curdlan to accelerate the expansion of *L. major*-specific CD4^+^ T cells has not been described yet. Thus, we isolated CD4^+^ T cells from SDLN of infected BALB/c mice 10 days after infection. BMDCs were pulse-infected with *L. major* parasites and incubated in the presence or absence of Curdlan. The incubation of CD4^+^ T cells with Curdlan-activated BMDCs resulted in a mild CD4^+^ T cell proliferation (Figures 8A,D). Proliferation of CD4^+^ T cells was substantially increased if BMDCs were pulsed with *L. major* parasites (Figures 8B,D). A pronounced enhancement of CD4^+^ T cell proliferation was achieved by activating *L. major*-infected BMDCs with Curdlan (Figures 8C,D). Consequently, Curdlan is capable of triggering the antigen-specific expansion of CD4^+^ T cells by infected BMDCs in a Dectin-1 specific manner (Figure S14 in Supplementary Material). Our data are in line with other experimental models showing that a Curdlan-induced priming of T cells depends on the Dectin-1 pathway (29). Curdlan also enhances the priming of antigen-specific T-cells by BMDCs exposed to *Leishmania* antigens (Figure S15 in Supplementary Material).

Supernatants of restimulated T cells were characterized by Multiplex analysis, to determine the expanded Th-cell subset. We could show that Curdlan treatment results in a 10-fold pronounced release of IFN-γ, IL-17, and IL-22 compared to control mice (Figure S16 in Supplementary Material). Thus, we conclude that Curdlan treatment induces a Th1 response in BALB/c mice that overlaps with a co-expression of Th17 cytokines (Figure S16 in Supplementary Material). Of note, the release of Th2 cytokines is not substantially modified (Figure S16 in Supplementary Material).

### A Mixture of *L. major* Antigens and Curdlan Induces a Milder Course of Experimental Leishmaniasis in BALB/c Mice

**Figure 9:**
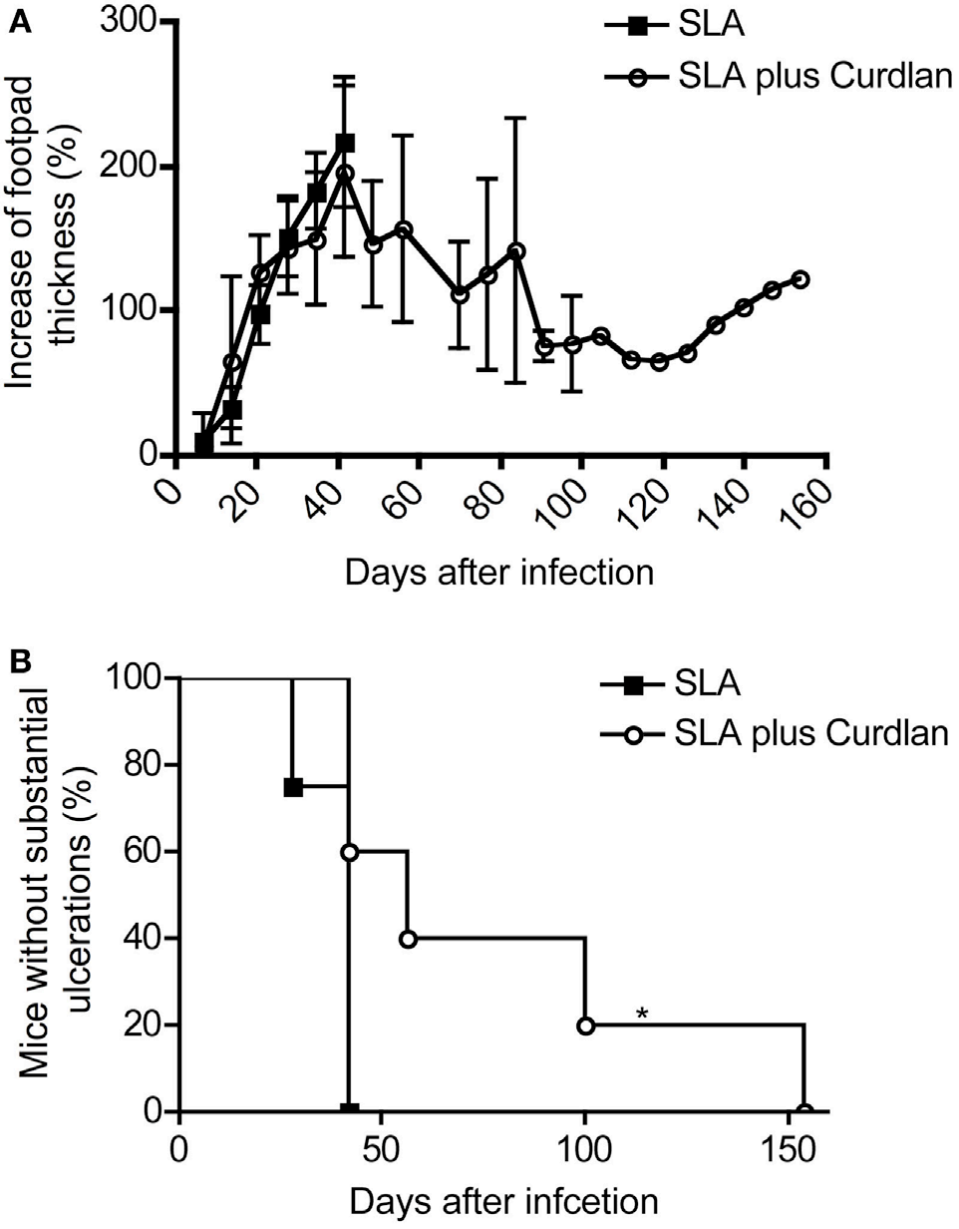
Course of disease in vaccinated mice challenged with *Leishmania (L.) major*. Soluble *L. major* antigens (SLA, black square) or SLA plus Curdlan (black circle) treated were injected intradermally into the left footpad of BALB/c mice. The contralateral right footpad was used for the intradermal infection with 3 × 10^6^ *L. major* parasites 28 days later. **(A)** The increase in footpad thickness after infection is shown (mean ± SD; *n* = 11 per group). **(B)** The number of mice that do not develop substantial ulcerations after intradermal infection into the contralateral hind footpad is depicted (*n* = 11; *p = 0.017). Significances were calculated using a log-rank test.

In combination with Curdlan, whole parasites might be used as a successful live “vaccine.” This immunization protocol protects BALB/c mice upon challenge with a high-dose reinfection from severe leishmaniasis (compare Figure 3). Consequently, protected BALB/c mice must have developed an adaptive T-cell memory response capable of controlling and eliminating *L. major* parasites (75). We investigated whether vaccination of BALB/c mice with *L. major* antigens (SLA) in combination with Curdlan might also be a promising approach. We tested the adjuvant potential of Curdlan by vaccinating mice intradermally with a mixture of SLA and Curdlan. 28 days later, mice were challenged by a high dose (3 × 10^6^) of *L. major* parasites into the contralateral footpad. Control mice that had received SLA in the absence of Curdlan developed a severe course of leishmaniasis that correlated with massive ulceration (compare Figure 3). These control groups were not protected, and the experiment had to be terminated at day 42 according to the animal health and welfare practices (Figures 9A,B).

These results again confirm the already published data that the simple administration of antigens does not induce protection of leishmaniasis in BALB/c mice (105). However, some BALB/c mice were partially protected after vaccination with SLA combination with Curdlan (Figure 9A). These mice developed a milder course of disease. Even though footpad swelling was still detectable, the ulceration of lesions was milder and substantially delayed (Figure 9B). Of note, a complete protection as achieved with living parasites could not be induced. The mice with delayed but ensuing mild ulcerations and constant footpad swelling were sacrificed during the monitoring period according to the guidelines for the care and use of experimental animals (Figure 9B). All analyzed lesions showing a delayed ulceration also exhibited a high parasite load (RPU > 1 × 10^8^).

## Discussion

Already in 1970s, β-glucans were used prophylactically or therapeutically in various infectious models of obligatory intracellular pathogens such as *Mycobacterium leprae* and *Plasmodium berghei* (106). Since β-glucans have no direct toxic effect on the named microbes, it has been suggested that a non-specific activation of the reticuloendothelial system was responsible for pathogen elimination (106). In experimental VL, β-glucans also elicited a strong but non-specific resistance against *L. donovani* in hamsters and BALB/c mice (107, 108). Other groups also demonstrated that an i.v. immunization with β-glucans combined with killed parasites induced a reduction in the parasite load in visceral organs of BALB/c and C57BL/6 mice (46, 109) and that CF-1 mice developed a milder course of VL, if they had been pretreated s.c. or i.v. with β-glucans in combination with killed *L. donovani* parasites (43, 110). These immunization experiments demonstrate that a combination of dead parasites and β-glucans supports the development of a protective host response in VL. A therapeutic effect of the β-glucan Curdlan has also been reported in *L. donovani* (MHOM/IN/1983/AG83)-infected BALB/c mice, in association with the generation of Th17 and Th1 cytokines if systemically (i.p.) treated with Curdlan (34).

In contrast to VL mouse models, the biological impact of β-glucans seems to be heterogeneous in experimental CL. For instance, a systemic application (i.v.) of β-glucans was not sufficient to modulate the immune response of C57BL/6 mice infected with various *Leishmania* species such as *L. mexicana, L. braziliensis*, and *L. garnhami* (45). On the other hand, *L. major* (MHOM/IL/80/Fredlin)-infected BALB/c mice develop a milder course of disease after multiple systemic applications (i.v. or i.p.) of β-glucans post infection (48). In line with the therapeutic potential of Curdlan, (i.p.) pretreatment with β-glucans dampened the spreading of *L. major* (HOM/SA/1983/SAYED) parasites in visceral organs of BALB/c mice (111). However, the effects of dermal applied β-glucans on the generation of adaptive T-cell responses had not been analyzed in detail.

In 2001, the CLR Dectin-1 was identified as the receptor for β-glucans (23). Subsequently, macrophages and professional antigen-presenting cells were identified as the most prominent cell subsets positive for Dectin-1 (112, 113). This finding raised the possibility that the activation of Dectin-1^+^ DC subsets during acquisition of adaptive immunity might be modulated by Dectin-1-dependent pathways. Based on the current model, Langerin^−^ dermal DCs are pivotal for the induction of the protective Th1-mediated immune response against *L. major* in experimental leishmaniasis (13, 15, 16). Thus, the precise conditioning of those DC subsets by Dectin-1-mediated signals might be crucial for the induction of adaptive immunity against *Leishmania* parasites. However, it is not known whether a cutaneous infection with *Leishmania* parasites causes an expansion of Dectin-1^+^ DCs in humans or experimental models. Additionally, it was not clear whether Dectin-1 signaling results in a pronounced expansion and polarization of *L. major*-specific T cells.

We showed that Dectin-1^+^ DC subsets expanded in patients suffering from CL. Consistent with other reports, we confirmed that Dectin-1 is expressed on peripheral blood mDCs of EHCs (27, 114). Furthermore, we were able to demonstrate that Dectin-1^+^ peripheral blood mDCs expand in patients suffering from CL compared to EHCs. Given that such an expansion of DC subsets might also take place at the site of infection and in SDLNs of CL patients, Dectin-1^+^ mDCs might represent promising targets for a Curdlan-based immunotherapy or vaccination strategy in humans. This is especially of interest since Dectin-1^+^ mDCs are discussed to decrease disease-promoting Th2-type responses (115). In contrast to mDCs, the expression of Dectin-1 by human pDCs is controversially discussed. Unlike mouse Dectin-1, human Dectin-1 is also expressed on B cells (27). Consequently, an expression of Dectin-1 on lymphoid cell subsets is quite conceivable. Indeed, it was demonstrated by Western blot analysis that Dectin-1 is expressed by human CD303^+^ (BDCA2) pDCs (116), whereas in another study CD303 (BDCA2)^+^ pDCs were reported mostly negative for Dectin-1 (117). It can be concluded that the frequency of Dectin-1^+^ DCs is higher within mDCs compared to pDCs. In line with these data, we could detect Dectin-1 on a small population of Lin^−^/CD11c^−^/CD123^+^ pDCs within the blood of EHCs and patients suffering from CL. Nevertheless, this extremely small population did not expand after infection. This is important for a Dectin-1 ligand-based vaccination or therapy because pDCs are supposed to favor disease-promoting Th2-type CD4^+^ T cell responses (118).

Infection with *Leishmania* parasites is associated with a pronounced myelopoiesis in mammalian hosts (119, 120). However, it has not been investigated so far whether this is also accompanied by an expansion of Dectin-1^+^ DCs. We show that Dectin-1^+^/CD11c^+^ DCs expand within the blood of infected BALB/c and C57BL/6 mice, as in patients suffering from CL. CD11c is hardly expressed by pDCs in mouse models (71, 72). Therefore, most CD11c^+/bright^ DCs belong to the subset of conventional DCs including other mDC subsets but not pDCs (73, 74). In addition, we found a clear increase in Dectin-1^+^/CD11c^+/bright^ DCs at the site of infection and SDLNs, proving the concept that Dectin-1^+^ DCs represent promising targets for a Curdlan-based immunotherapy.

*In vitro* analysis was performed to characterize such a potentially beneficial impact of Curdlan/Dectin-1 interactions on DC-mediated priming of CD4^+^ Th cells. To accomplish these tasks, DCs must undergo maturation to express surface molecules pivotal for migration such as CCR7 and costimulatory molecules (14, 17). Previous studies have already demonstrated that Curdlan can induce DC maturation (121, 122), whereas the biological effect of Curdlan on *Leishmania*-infected DCs was unknown. Parasite harboring DCs show an enhanced Dectin-1 expression but are immature. Curdlan activation resulted in the maturation of parasite-harboring DCs and the release of DC-activating cytokines. Subsequently, Curdlan-primed infected DCs were more potent in expanding antigen-specific T cells compared to DCs that had not been primed with Curdlan. Of note, Dectin-1^−/−^ mice (on BALB/c background) were not protected from severe leishmaniasis by dermal Curdlan application. Additionally, Curdlan stimulation could neither mature BMDCs from Dectin-1^−/−^ mice nor enhance the priming of antigen-specific T cells. These findings clearly demonstrate that Dectin-1 is exclusively responsible for the Curdlan-mediated effects *in vivo* and *in vitro*. These data are in line with the already published studies, demonstrating a Dectin-1-specific adjuvant effect of Curdlan on T cell priming in other experimental models (29, 98, 123). Consequently, Dectin-1 ligation can be considered as an important checkpoint for DC maturation and functionality in leishmaniasis.

We would like to point out that surface Dectin-1 is predominantly expressed by immature DC. This explains why infected DCs that in turn display an immature phenotype show a higher Dectin-1 mean fluorescence intensity (MFI) compared to uninfected DCs. Comparable to macrophages, Curdlan-exposed DCs respond with a reduction in the MFI of surface Dectin-1. Whether this reduction is due to the Dectin-1/Curdlan complex internalization, as described for macrophages, is not clear (124). Given the fact that mature DCs in general express lower amounts of surface Dectin-1, it cannot be excluded that maturation of DCs in general is accompanied by the downregulation of surface Dectin-1. In the light of our results, the data from Lima-Junior et al. deserves special consideration. The authors show an enhanced Dectin-1 MFI in *L. amazonensis* infected macrophages (33). Given the fact that infected and immature DCs display also a higher surface Dectin-1 MFI, it can be suggested that *Leishmania* parasites might favor preferentially Dectin-1^+^ myeloid cells. Indeed, Dectin-1^−/−^ phagocytes respond with a moderate reduction in the uptake of *L. infantum* parasites (31). However, we found that Dectin-1-Fc fusion proteins do not bind to *L. major* parasites. Thus, a *L. major/*Dectin-1 interaction can be excluded. This is of crucial importance in three aspects: first, *L. major* parasites do not induce Dectin-1 signaling-associated maturation of DCs, which might be considered as a smart escape mechanism, preventing DC maturation and subsequent T-cell activation. Second, the higher frequency of infected Dectin-1-positive DCs is not the result of *L. major* Dectin-1 interactions, but might be an upregulation, based on so far not known mechanisms (33). Third, these data might explain the finding that Dectin-1^−/−^ phagocytes can still phagocyte *L. infantum* parasites (31).

Apart from these items discussed earlier, Dectin-1^+^ mDCs might represent useful targets for the induction of a protective Th1-polarized CD4^+^ T-cell response by β-glucans. Indeed, *in vivo* experiments revealed that a dermal application of Curdlan in the presence of living parasites induced a resistant phenotype in normally susceptible BALB/c mice. It is known that CLR signaling affects macrophage function in experimental leishmaniasis (31). Thus, we had to demonstrate in the high-dose infection model that the Curdlan-mediated effect on protective T-cell immunity was not the result of an early elimination of parasites at the site of infection by dermal macrophages. This would otherwise lead to a substantial reduction in parasite numbers shortly after high-dose infection. This is of crucial importance because low-dose infected BALB/c mice can mount a protective Th1 response (8, 125). Since the early parasite load at the site of infection was unaltered in the presence of Curdlan, we can exclude Curdlan-mediated early effects on *L. major* clearance by phagocytes. This finding was supported by the fact that Curdlan did not induce leishmanicidal mechanisms in infected macrophages *in vitro*. Hence, protection in Curdlan-treated BALB/c mice is not mediated by unspecific innate immune mechanisms, but an adaptive T cell-mediated response.

The ratio of Th1/Th2 cell and cytokines is crucial for efficient T cell-mediated immune responses and the outcome of leishmaniasis (86). From our data we can conclude that Curdlan treatment elicits a clear shift toward a protective Th1-mediated response in normally susceptible BALB/c mice. This conclusion is justified because Curdlan-treated BALB/c mice developed a resistant phenotype and show a clear DTH response that is associated with the generation of a T cell memory response, sufficient to protect BALB/c mice from a high-dose reinfection (75). The pronounced *Leishmania*-specific IgG2/IgG1 ratio supports also a manifested *Leishmania*-specific Th1 response (3). Furthermore, CD4^+^ T cells isolated from SDLNs of Curdlan-treated infected BALB/c mice produced a lower level of IL-4 mRNA, but an increased level of cytokines such as IFN-γ and TNF-α after antigen-specific restimulation, further supporting a Th1 shift. These data are in line with previous studies, showing that early IL-4/IL-4R interactions promote Th2-cell polarization and impair the development of a Th1 response after infection with *L. major* (7, 85, 126–131). The enhanced release of IL-22 and IL-17 by CD4^+^ T cells of Curdlan-treated mice needs a closer view. In mice, IL-17 and IL-22 are preferentially associated with Th17-cell differentiation (132). This is of interest, because the expressions of IL-17 and IL-22 are supposed to be crucial cofactors for *Leishmania* vaccine-induced immunity (133–136).

Successful vaccination programs against *Leishmania* parasites are still problematic. However, whole-organism vaccination strategies are most promising (137, 138). This implicates that pathogen-associated inflammatory responses at the site of infection might be crucial for the recruitment of appropriate immune cells and the differentiation of antigen-presenting cells, thus resulting in protective immunity (14, 139). Whole organisms in combination with Curdlan induce a protective immune response in normally susceptible BALB/c mice. The classical “vaccination” by *Leishmania* antigens in combination with Curdlan was able to dampen the severe progress of disease. However, full protection could not be achieved. The failure to mount a profound and solid adaptive immunity might be explained by the fact that *Leishmania* antigens do not persist at the site of infection and do not induce inflammatory immune responses, crucial for driving Th1-mediated adaptive immunity. In conclusion, we propose that whole-organism application in combination with Curdlan represents a promising strategy for the induction of *Leishmania-*specific immunity initiated by Dectin-1^+^ DCs.

## Ethics Statement

Animals: all experiments and animal housing were performed according to the guidelines for the care and use of experimental animals. The animal work was approved by the local veterinary authorities of the district government based on the European guidelines and national regulations of the German Animal Protection Act (approval no. AZ 54-2532.1-05/11). Humans: the sample collection was permitted based on the local ethical committee (allowance number PO25/08, Addis Ababa, Ethiopia) and the national health research ethics review committee (approval number 310/227/07, Addis Ababa). Written informed consent was obtained from the participants of this study.

## Author Contributions

NZ acquired most of the data and analyzed the data. JJ and VS performed the macrophage experiments. MC performed the flow cytometry analysis in Ethiopia, AA, GZ, BL, MS, RW, AR, AW, and GB made substantial contributions to conception of the project and the article. GB and BL contributed materials essential for the work. UR designed the project and drafted and revised the manuscript critically for intellectual content.

## Conflict of Interest Statement

The authors declare that the research was conducted in the absence of any commercial or financial relationships that could be construed as a potential conflict of interest.
