# Supplementary material for: Dectin-1 Positive Dendritic Cells Expand after Infection with Leishmania major Parasites and Represent Promising Targets for Vaccine Development
Source: Front Immunol. 2018 Feb 26;9:263. doi: 10.3389/fimmu.2018.00263 (PMC5834765; doi:10.3389/fimmu.2018.00263)
Supplement: Supplementary file 1 [file data_sheet_1.pdf]

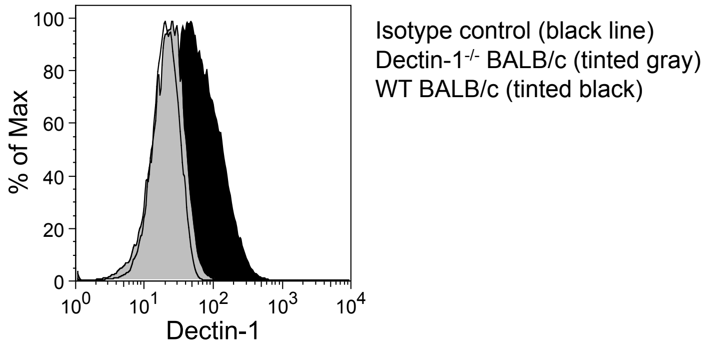

Supplementary Figure 1: Detection of Dectin-1 on BMDCs. BMDCs from Dectin-$1^{-/-}$ and wild type (WT) BALB/c mice were stained for CD11c and Dectin-1. The histogram visualizes the specificity of the Dectin-1-antibody. Cells were gated on $CD11c^+$ BMDCs.

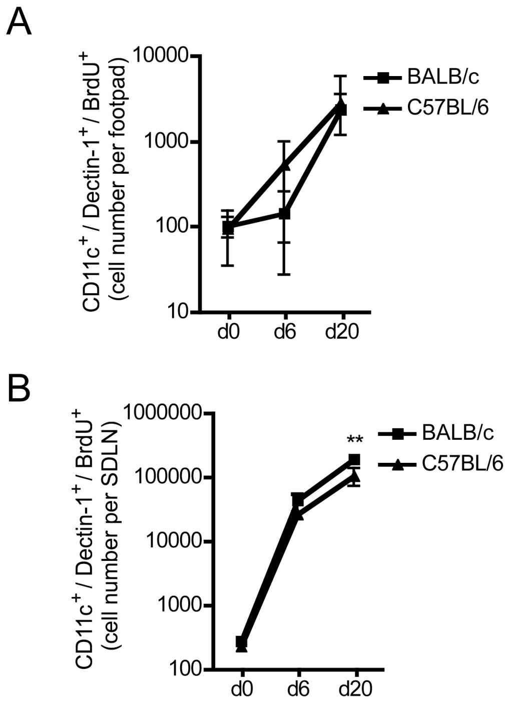

Supplementary Figure 2: Characterization of Dectin-1 expression on proliferating $CD11c^+$ DCs at the site of infection and SDLNs of infected C57BL/6 and BALB/c mice. Mice were infected with *L. major* parasites and BrdU was given 3 days before analysis. The indicated tissues were analyzed at day 6 and day 20 after infection. Mice that have not been infected with *L. major* parasites represent the controls (day 0). The same gating strategy as displayed in Figure 2 A-C was used. Graphs represent the increase of $BrdU^+/CD11c^+$ DCs at the site of infection (A) and SDLNs (B) of BALB/c (square) and C57BL/6 (triangle) mice (mean ±SD). Pooled data from n=3 different experiments are depicted. Data were analyzed using 2way ANOVA (**p = 0.01).

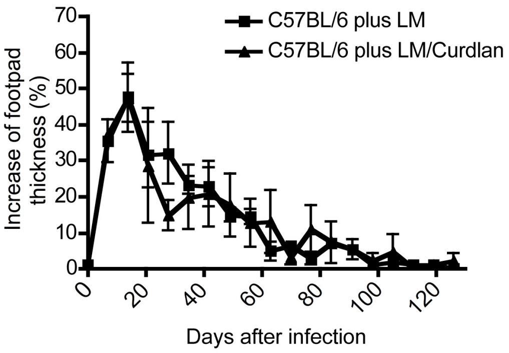

Supplementary Figure 3: The presence of Curdlan at the site of infection does not accelerate the course of experimental leishmaniasis in resistant C57BL/6 mice. C57BL/6 control mice were infected with $3x10^6$ *L. major* parasites (black squares; n=3). Another group was infected with 30 μL of a mixture of $3x10^6$ parasites and Curdlan (c = 50μg/μL, n=3, black triangles). The y-axis depicts the increase of footpad thickness (mean ±SD) and the x-axis days after infection.

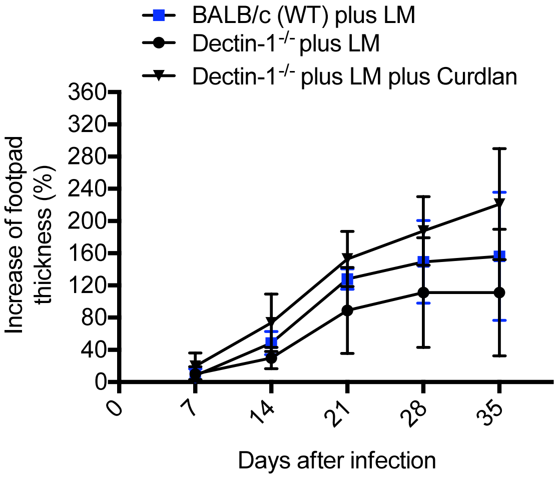

Supplementary Figure 4: The presence of Curdlan at the site of infection does not change the course of leishmaniasis in BALB/c mice deficient for Dectin-1. BALB/c wild type (WT, blue squares; n=5) and Dectin-1$^{-/-}$ mice were infected with $3x10^6$ *L. major* parasites in the presence (black circle; n=5) or absence of Curdlan (black triangle; n=5). The y-axis depicts the increase of footpad thickness (mean ±SD) and the x-axis days after infection.

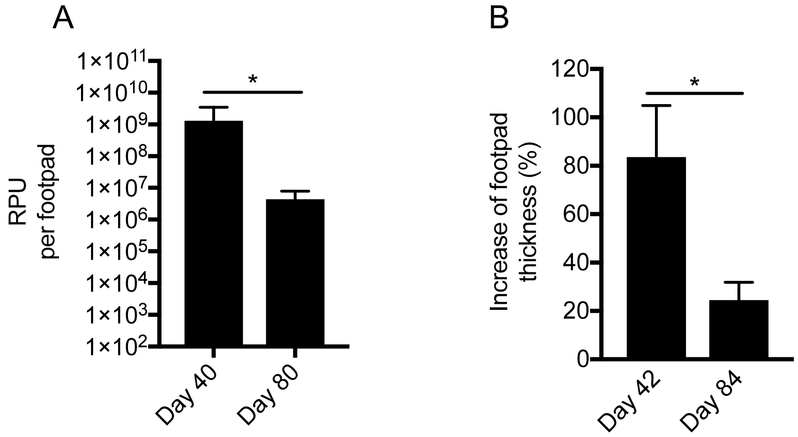

Supplementary Figure 5: Characterization of the parasite burden of Curdlan treated BALB/c mice. BALB/c mice were infected with 30 µl of a mixture of $3x10^6$ parasites and Curdlan (c=50µg/µL). A) Relative parasite units (RPU) representing the relative amount of parasites per footpad at day 40 (n=6) and 80 (n=6) after infection are displayed. Data were analyzed using the nonparametric Mann-Whitney test (*p = 0.038). B) The representative increase of footpad thickness is displayed. Pooled data from three independent experiments were analyzed using the nonparametric Mann-Whitney test (day 42 n=15, day 84 n=11, *p= 0.034). The mean ±SD are shown.

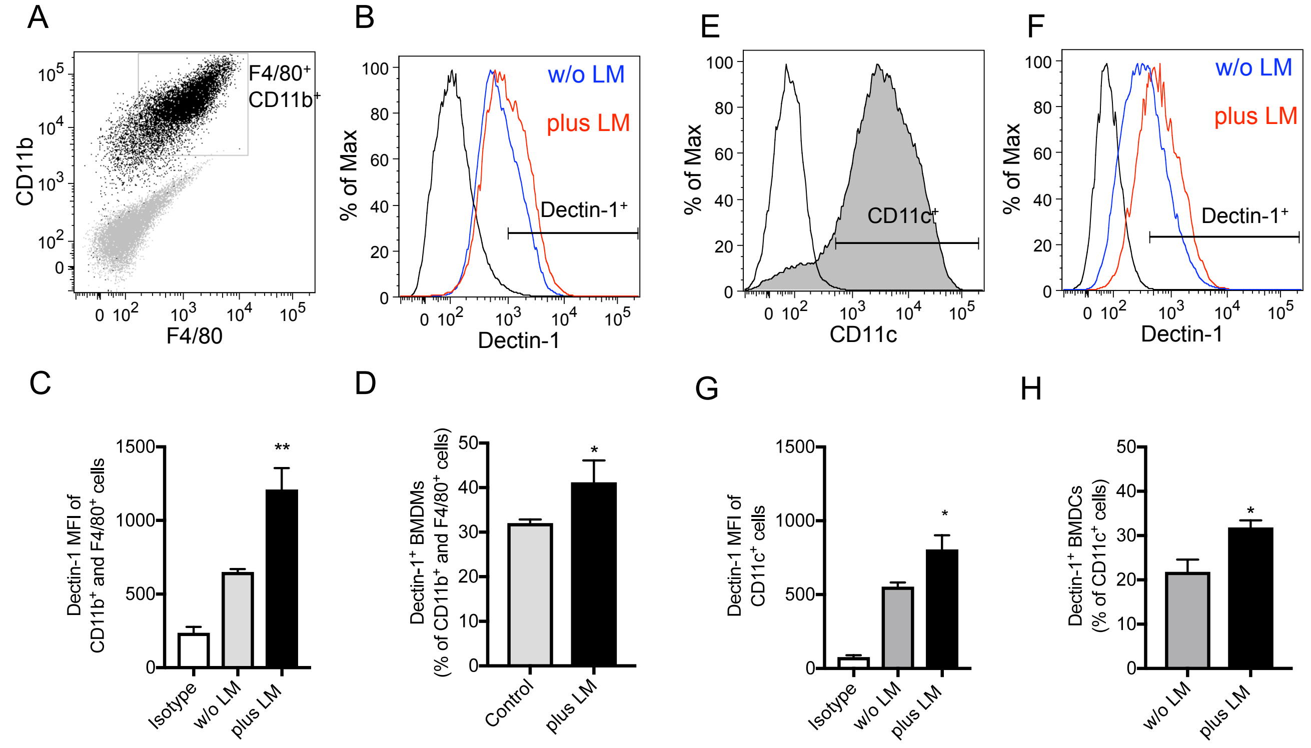

Supplementary Figure 6: Dectin-1 expression by myeloid cell subsets in the presence and absence of *L. major* parasites. BMDMs and BMDCs were infected with *L. major* promastigote parasites (5 parasites : 1 myeloid cell). After 24 hours the cells were harvested and characterized by flow cytometry analysis for the markers CD11b, F4/80, CD11c and Dectin-1. A) The dot plot diagram depicts $F4/80^+$/ $CD11b^+$ BMDMs (black dots). The gate was placed according the isotype controls (gray dots). B) The histogram visualizes the expression of Dectin-1 of *L. major* (LM) infected cultures (red line, plus LM) and not infected cultures (blue line, w/o LM) BMDCs. C) The mean fluorescence intensity (MFI) of Dectin-1 expressed by $F4/80^+$/$CD11b^+$ BMDMs is shown (*p=0.027, n=3). D) The frequency of Dectin-1 positive $F4/80^+$/$CD11b^+$ BMDMs cultures (not infected = control and infected = plus LM) is displayed (n=3 experiments, *p=0.003). E) The histogram depicts $CD11c^+$ BMDCs (tinted black) and the representative isotype controls (gray line). B) The histogram visualizes the expression of Dectin-1 of *L. major* (LM) infected BMDC cultures (red line, plus LM) and not infected BMDM cultures (blue line, w/o LM) BMDCs. C) The mean fluorescence intensity (MFI) of Dectin-1 expressed by $CD11c^+$ BMDCs is shown. (**p=0.0042, n=3). D) The frequency of Dectin-1 positive $CD11c^+$ cultures (w/o LM = control and infected = plus LM) is displayed (n=3 experiments, *p=0.0128). Data were analyzed using the nonparametric Mann-Whitney test and are representative for two independent experiments.

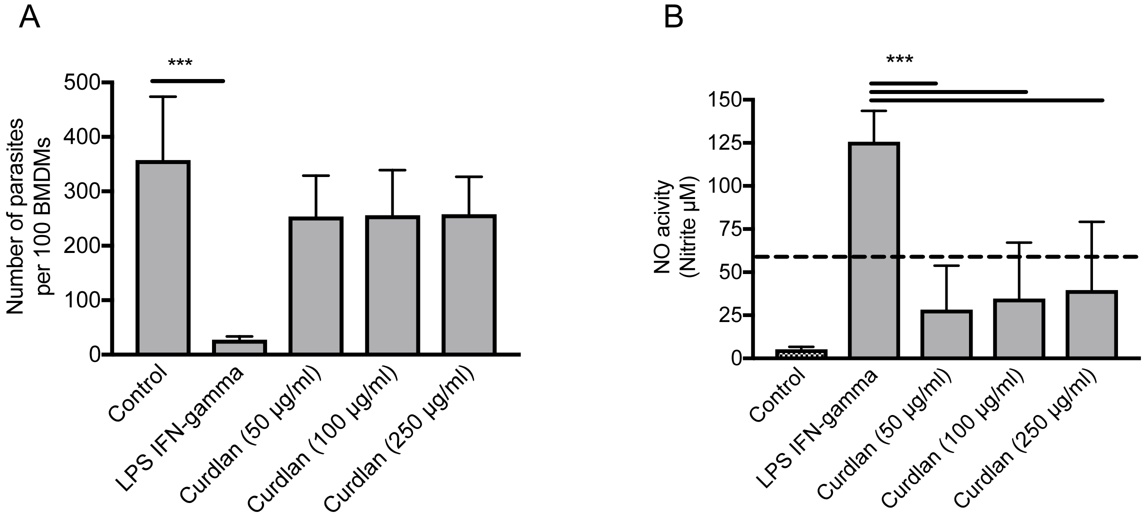

Supplementary Figure 7: BMDMs cannot be stimulated by Curdlan to eliminate intracellular *L. major* parasites. BMDM were puls-infected (1:30 over 4h) with promastigote parasites and stimulated with LPS/IFN-gamma (10 ng/ml and 20 ng/ml) or Curdlan (50 µg/ml, 100 µg/ml, 250 µg/ml). 72 hours later the clearance of parasites was determined and NO activity was determined. A) The number of parasites per 100 BMDMs was determined by Giemsa staining. Pooled data from two independent experiments are shown. The Data were analyzed using Student's t-test (n=6 per group; ***p<0.001). B) NO activity (Nitrite µM) was measured by the Griess reaction. Pooled data from two independent experiments are shown. The dotted horizontal line depicts the threshold of NO activity necessary to induce leishmanicidal effects. Data were analyzed using Student's t-test (n=6 per group; ***p<0.001). The mean ±SD are shown.

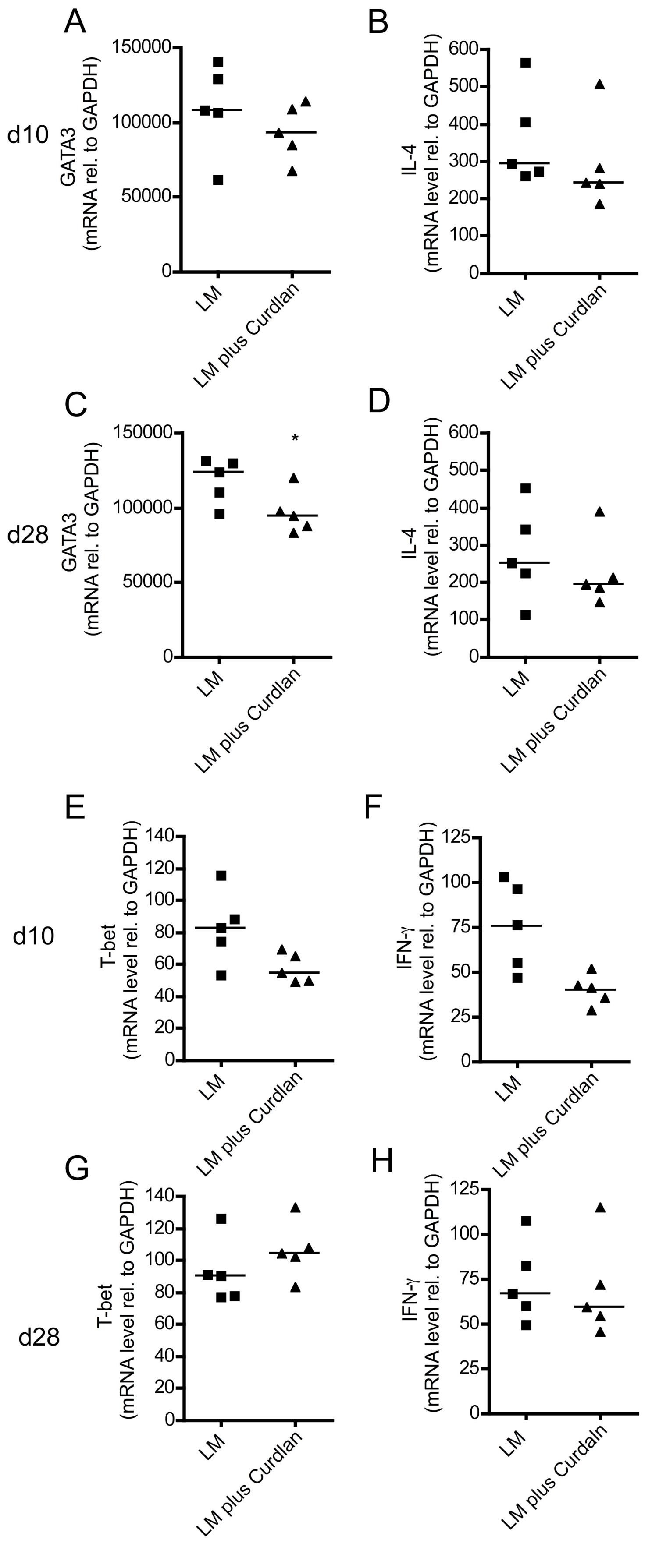

Supplementary Figure 8: Lymph node resident CD4$^+$ T cells of Curdlan treated BALB/c mice show a dampened GATA3 and IL-4 expression. BALB/c mice were infected with a volume of 30 µL PBS containing 3x10^6 *L. major* (LM) parasites in the presence (plus Curdlan, c = 0 µg/µL) or absence of Curdlan. CD4$^+$ T cells were purified at day 10 and 28 after infection. qRT-PCR analysis was performed to determine the relative mRNA levels of target genes referred to GAPDH. The relative expression of GATA-3 and IL-4 at day 10 (A and B) and 28 (C and D) after infection (n=5; *$p<0.05$) and the relative expression of T-bet and IFN-γ at day 10 (E and F) and 28 (G and H) after infection are displayed. Each symbol represents an individual mouse and the bars indicate the medians. Data were analyzed using Student's t-test.

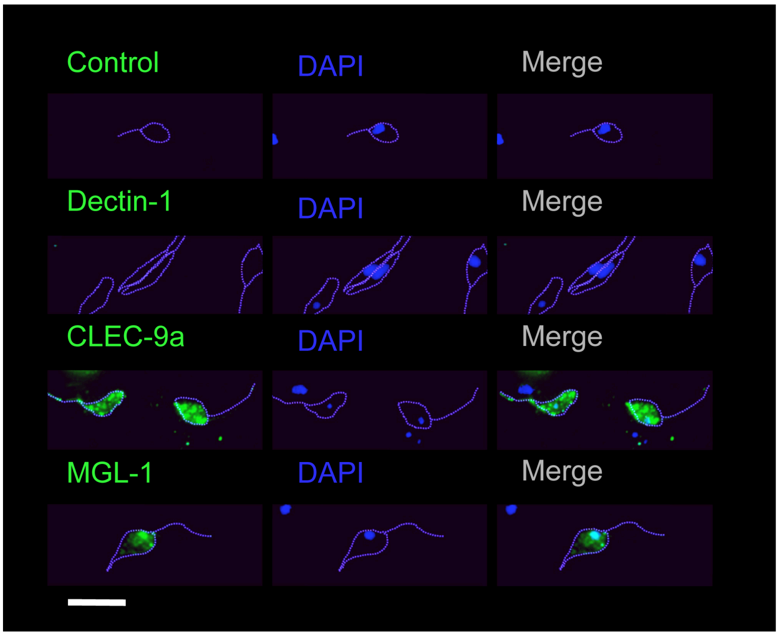

Supplementary Figure 9: Detection of selected pathogenic ligands by CLR-Fc fusion proteins. Promastigote parasites were stained with the indicated CLR-Fc fusion proteins. Human IgG1 was used as control. CLR-Fc binding to *L. major* was detected with an AF488-conjugated goat anti-hFc antibody. *Leishmania* DNA is visualized by DAPI. One out of two independent experiments is displayed (bar 10 µm).

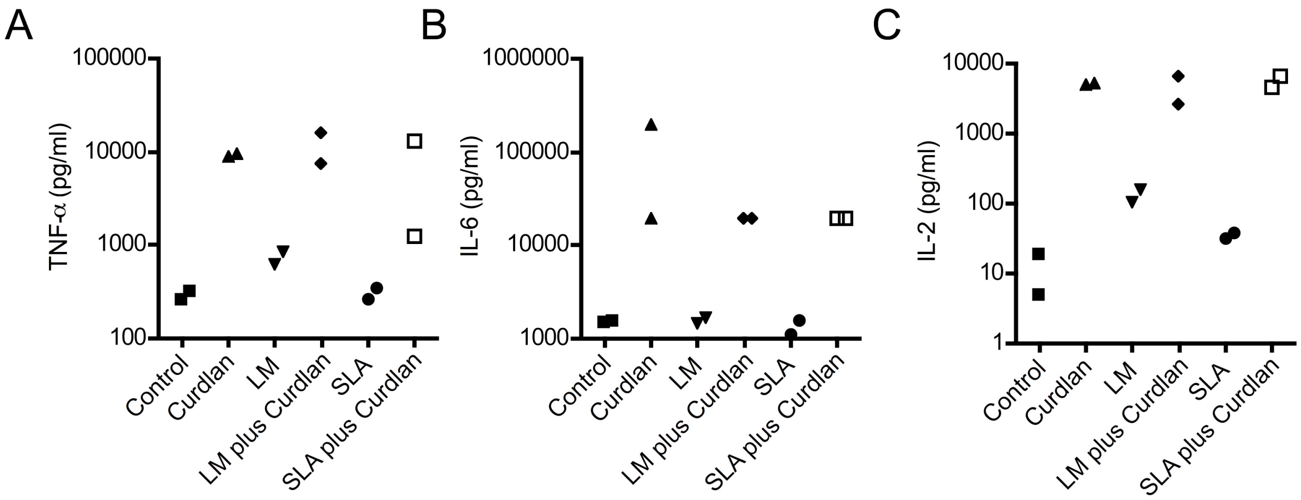

Supplementary Figure 10: Curdlan stimulation results in a pronounced cytokine secretion of infected BMDCs. BMDCs were cultured for 24 hours under the following conditions: not infected BMDCs (Control); not infected BMDCs plus Curdlan (Curdlan); *L. major* infected BMDCs (LM); *L. major* infected BMDCs in the presence of Curdlan (LM plus Curdlan); BMDCs plus soluble *L. major* antigens (SLA); BMDCs plus *L. major* antigens in the presence of Curdlan (SLA plus Curdlan). The cytokines TNF-α, IL-6 and IL-2 were determined by multiplex analysis. Two pooled samples from 3 experiments were analyzed.

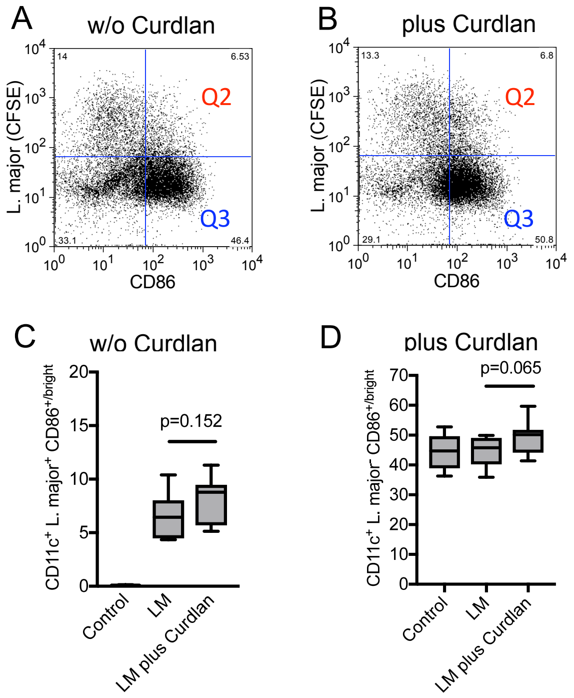

Supplementary Figure 11: *L. major* harboring Dectin-1$^{-/-}$ BMDCs do not mature after Curdlan stimulation. Dectin-1$^{-/-}$ BMDCs from BALB/c mice were incubated with CFSE-labeled *L. major* promastigote parasites in the presence or absence of Curdlan. After 24 hours Dectin-1$^{-/-}$ BMDCs were harvested and characterized by flow cytometry using the markers CD11c and CD86. A) The dot plot diagram depicts four subsets (see quadrants) of Dectin-1$^{-/-}$ BMDCs incubated with *L. major* parasites in the absence of Curdlan and B) in the presence of Curdlan. The quadrants of interest are upper right (*L. major* CFSE^{+}/CD86$^{+/bright}$; Q2) and lower right (*L. major* CFSE^{-}/CD86$^{+/bright}$; Q3). The quadrants placed according the isotype controls of not infected BMDCs. C) The frequency of *L. major* CFSE^{+} and CD86$^{+/bright}$ BMDCs (Q2) is shown. D) The frequency of *L. major* CFSE^{-} and CD86$^{+/bright}$ (Q3) BMDCs is depicted. The following culture conditions have been compared: Not infected Dectin-1$^{-/-}$ BMDCs (Control), *L. major* harboring Dectin-1$^{-/-}$BMDCs (LM), and *L. major* harboring Dectin-1$^{-/-}$ BMDCs stimulated with Curdlan (LM plus Curdlan). Data were analyzed using the nonparametric Mann-Whitney test (n=8; C: p=0.152 and D: p=0.065).

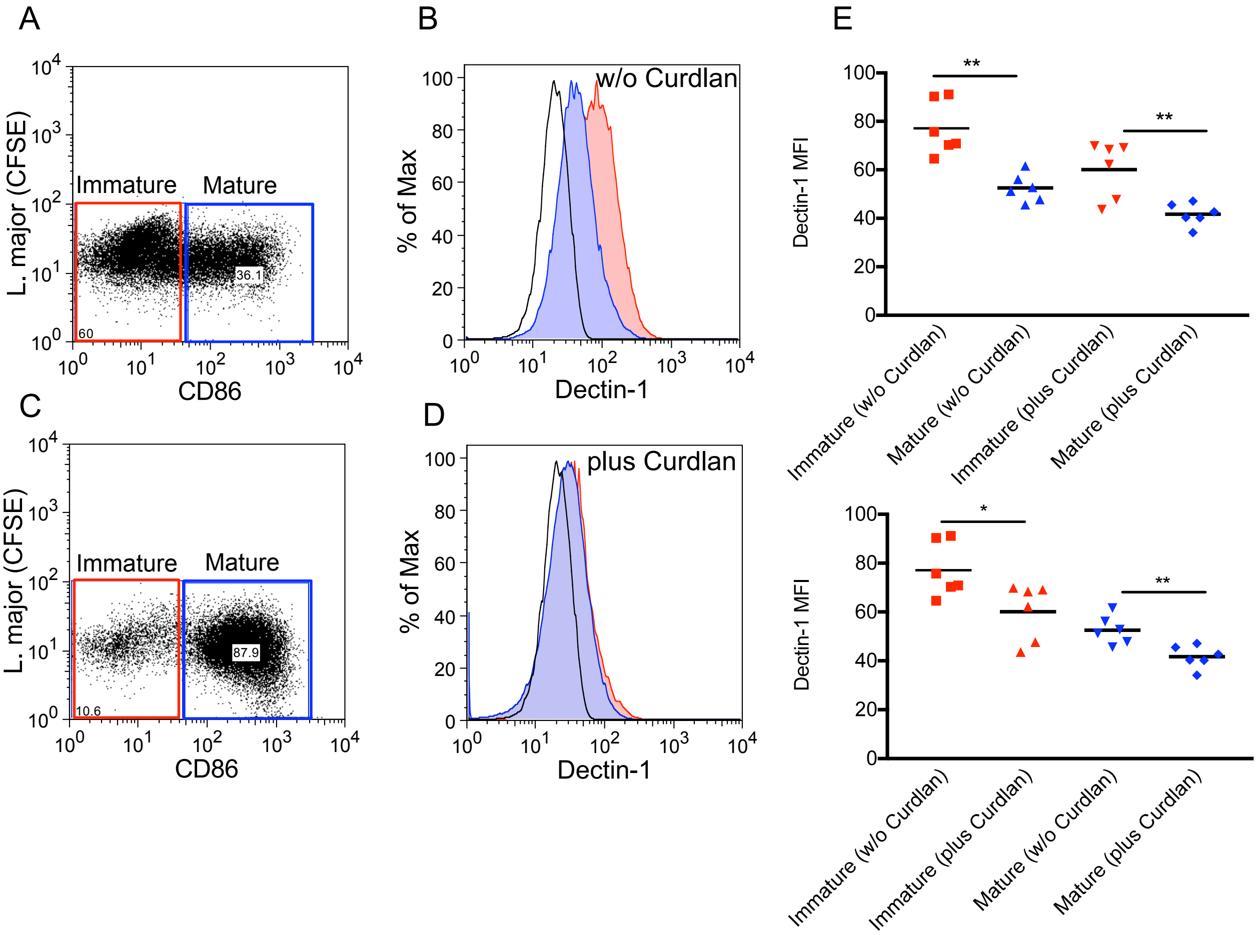

Supplementary Figure 12: Curdlan activation results in a reduction of the Dectin-1 expression by BMDCs. BMDCs were incubated in the presence or absence of Curdlan. BMDCs that were not exposed to parasites were used as controls. A) BMDCs that have note been exposed to CFSE-labeled *L. major* promastigote parasites, were harvested and characterized by flow cytometry using the markers CD86, CD11c and Dectin-1. The dot plot diagram depicts CD86$^{low/-}$ immature (red square) and mature (CD86$^{+/bright}$, blue square) CD11c^{+} BMDCs. B) The MFI of Dectin-1^{+} cells within the indicated subsets (tinted red = immature, tinted blue = mature, black line = isotype control) is shown by an histogram. C) The dot plot diagram depicts BMDCs that have been stimulated with Curdlan. D) The Dectin-1 MFI is shown with a histogram. CD86$^{low/-}$ immature (red square) and mature (CD86$^{+/bright}$, blue square) CD11c^{+} BMDCs were analyzed (tinted red = immature, tinted blue = mature, black line = isotype control). E) The scatter plots depicts the quantification of the Dectin-1 MFI on CD11c^{+} BMDCs stimulated (plus Curdlan) or unstimulated (w/o Curdlan). (Upper plot. **p= 0.002, **p=0.009. Lower plot *p=0.015, **p=0.004. The horizontal line represents the mean, the symbols independent sample. The data are representative for three experiments.

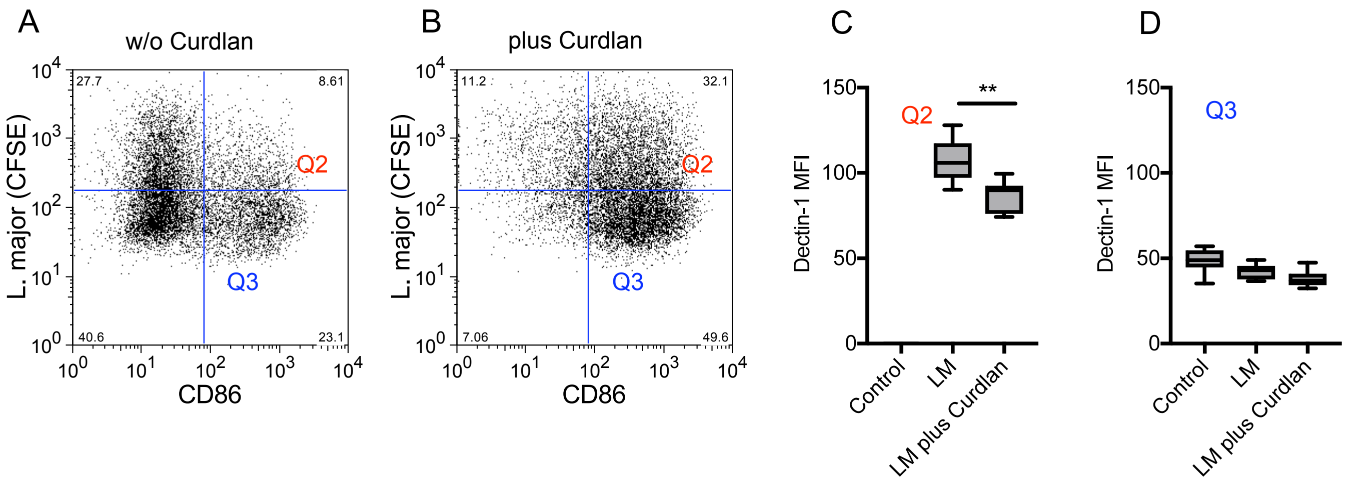

Supplementary Figure 13: Curdlan activation results in a reduction of the Dectin-1 expression by *L. major* harboring BMDCs. BMDCs were incubated with CFSE-labeled *L. major* promastigote parasites in the presence or absence of Curdlan. BMDCs that were not exposed to parasites were used as controls (compare Figure 7A). A) The dot plot depicts CD11c^{+} BMDCs incubated with CFSE-labeled *L. major* promastigote parasites in the absence of Curdlan (w/o Curdlan). The quadrants of interest are upper right (*L. major* CFSE^{+}/CD86$^{+/\mathrm{bright}}$; Q2) and lower right (*L. major* CFSE^{-}/CD86$^{+/\mathrm{bright}}$; Q3). The quadrants were placed according the isotype controls of not infected BMDCs. B) The dot plot depicts CD11c^{+} BMDCs incubated with CFSE-labeled *L. major* promastigote parasites in the presence of Curdlan (plus Curdlan). C) The frequency of *L. major* CFSE^{+} and CD86$^{+/\mathrm{bright}}$ BMDCs (Q2) is shown. D) The frequency of *L. major* CFSE^{-} and CD86$^{+/\mathrm{bright}}$ (Q3) BMDCs is depicted. The following culture conditions have been compared: Not infected BMDCs (Control), *L. major* harboring BMDCs (LM), and *L. major* harboring BMDCs stimulated with Curdlan (LM plus Curdlan). The box plots depict pooled data out of two independent experiments (n=6). Data were analyzed using the nonparametric Mann-Whitney test (C, **p=0.005).

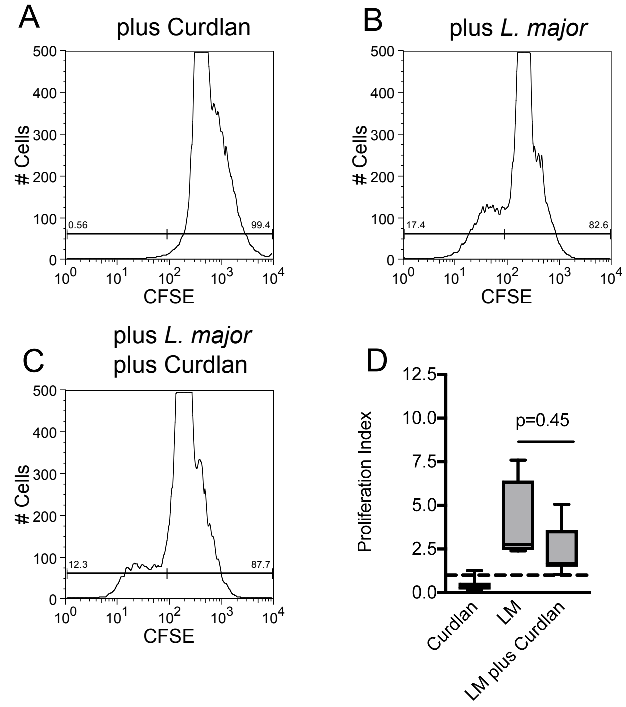

Supplementary Figure 14: Curdlan stimulation does not enhance the potential of parasite harboring Dectin-1$^{-/-}$ BMDCs towards the expansion of *Leishmania*-specific CD4^{+} T cells. Dectin-1$^{-/-}$ BALB/c mice were infected with a volume of 30 µL PBS containing 3x10^{6} *L. major* (LM) parasites. Ten days after infection CD3^{+} T cells were isolated from SDLNs and labeled with CFSE. Dectin-1$^{-/-}$ BMDCs were harvested at day ten after *ex vivo* differentiation with GM-CSF and incubated for 24 hours with *L. major* parasites (5:1) in the presence or absence of Curdlan (50µg/200µL). CFSE-labeled Dectin-1$^{-/-}$ CD3^{+} T cells and Dectin-1$^{-/-}$ BMDCs (10:1) were incubated for 72h and analyzed by flow cytometry. The proliferative (left gate) and resting (right gate) CD4^{+} T cells are visualized by gates within the histogram plots. Following experimental approaches are displayed: A) Curdlan activated but not infected Dectin-1$^{-/-}$ BMDCs (plus Curdlan), B) Infected but not activated Dectin-1$^{-/-}$ BMDCs (plus *L. major*), and C) Curdlan activated and infected Dectin-1$^{-/-}$ BMDCs (plus *L. major,* plus Curdlan). The percentage of resting and proliferating cells is indicated inside of the corresponding gates. D) The proliferation Index is displayed as a box plot diagram. The horizontal line visualizes the background proliferation of unstimulated BMDCs and CD4^{+} T cells. The Data were analyzed using Student's t-test (n=6 per group; p=0.45).

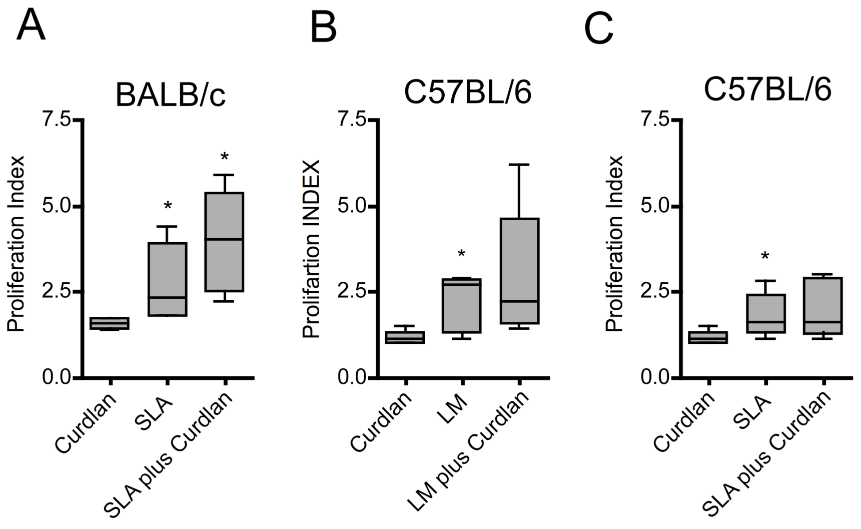

Supplementary Figure 15: Characterization of *Leishmania*-specific T cell proliferation in response to BMDCs that have been infected with *L. major* or primed with parasite antigens. BALB/c and C57BL/6 mice were infected with a volume of 30 µL PBS containing 3 x10^6 *L. major* (LM) parasites. Ten days after infection $CD3^+$ T cells were isolated from SDLNs and labeled with CFSE. BMDCs were harvested at day 10 after differentiation with GM-CSF and pulse-infected for 24 hours with *L. major* parasites (5:1) in the presence or absence of Curdlan (50µg/200µL). CFSE-labeled $CD3^+$ T cells and BMDCs (10:1) were incubated for 72 hours and analyzed with the flow cytometer. The proliferative responses of $CD4^+$ T cells were calculated (compare Figure 8). The experimental approaches are shown as box plots: A) BALB/c mice: Curdlan activated (Curdlan), *L. major*-antigen primed BMDCs (SLA), and *L. major*-antigen primed and Curdlan activated BMDCs (SLA plus Curdlan) (n=5 per group, $*p^{SLA}$=0.029 and $*p^{SLA\ plus\ Curdlan}$=0.032). B) C57BL/6 mice: Curdlan activated (Curdlan), *L. major* infected BMDCs (LM), and *L. major* infected and Curdlan activated BMDCs (LM plus Curdlan) (n=5 per group, *p=0.033). C) C57BL/6 mice: Curdlan activated (Curdlan), *L. major*-antigen primed BMDCs (SLA), and *L. major*-antigen primed and Curdlan activated BMDCs (SLA plus Curdlan) (n=5 per group, *p=0.049). Data were analyzed using Student's t-test.

	Th1	Th2	Th17
IFN-γ	12		
TNF-α	5		
IL-4		1	
IL-5		2	
IL-6		8	
IL-13		4	
IL-17			14
IL-21			2
IL-22			>15

1
2
3
4
5
6
7
8
9
10
11
12
13
14
>15

Supplementary Figure 16: Multiplex analysis of cytokines: BALB/c mice were infected with 3 x10^6 *L. major* (LM) parasites. Ten days after infection CD3$^+$ T cells were isolated from SDLNs. BMDCs were harvested at day ten after differentiation with GM-CSF and pulse infected 24 hours with *L. major* parasites (5:1) in the presence or absence of Curdlan (50µg/200µL). CD3$^+$ T cells and BMDCs (10:1) were incubated for 72h. Supernatants were collected and a Multiplex analysis was performed. The heat map illustrates the x-fold increase of cytokines in *L. major* infected BMDCs compared to *L. major* infected BMDCs plus Curdlan activation. One representative experiments is shown.
